# Crystal structures of bis­(phen­oxy)silicon phthalocyanines: increasing π–π inter­actions, solubility and disorder and no halogen bonding observed

**DOI:** 10.1107/S205698901600935X

**Published:** 2016-06-21

**Authors:** Benoît H. Lessard, Alan J. Lough, Timothy P. Bender

**Affiliations:** a University of Toronto, Department of Chemical Engineering & Applied Chemistry, 200 College Street, Toronto, Ontario, M5S 3E5, Canada; b University of Ottawa, Department of Chemical and Biological Engineering, 161 Louis Pasteur, Ottawa, Ontario, K1N 6N5, Canada; c University of Toronto, Department of Chemistry, 80 St. George Street, Toronto, Ontario, M5S 3H6, Canada; d University of Toronto, Department of Materials Science and Engineering, 200 College Street, Toronto, Ontario, M5S 3E5, Canada

**Keywords:** crystal structure, silicon, phthalocyanine, phenol, phen­oxy, phen­oxy­lation, inter­actions, halogen, bonds

## Abstract

We report the syntheses and characterization of three solution-processable phen­oxy silicon phthalocyanines (SiPcs), namely bis­(3-methyl­phen­oxy)(phthalocyanine)silicon [(3MP)_2_-SiPc], C_46_H_30_N_8_O_2_Si, bis­(2-*sec*-butyl­phen­oxy)(phthalocyanine)silicon [(2*sec*BP)_2_-SiPc], C_44_H_24_I_2_N_8_O_2_Si, and bis­(3-iodo­phen­oxy)(phthalocyanine)silicon [(3IP)_2_-SiPc], C_52_H_42_N_8_O_2_Si. Crystals grown of these compounds were characterized by single-crystal X-ray diffraction and the π–π inter­actions between the aromatic SiPc cores were studied. It was determined that (3MP)_2_-SiPc has similar inter­actions to previously reported bis­(3,4,5-tri­fluoro­phen­oxy)silicon phthalocyanines [(345 F)_2_-SiPc] with significant π–π inter­actions between the SiPc groups. (3IP)_2_-SiPc and (2*sec*BP)_2_-SiPc both experienced a parallel stacking of two of the peripheral aromatic groups. In all three cases, the solubility of these mol­ecules was increased by the addition of phen­oxy groups while maintaining π–π inter­actions between the aromatic SiPc groups. The solubility of (2*sec*BP)_2_-SiPc was significantly higher than other bis-phen­oxy-SiPcs and this was exemplified by the higher observed disorder within the crystal structure.

## Abstract 2

We report the syntheses and characterization of three solution-processable phen­oxy silicon phthalocyanines (SiPcs). The π–π inter­actions between the aromatic SiPc cores were studied. In all three cases, the solubility of the mol­ecules was increased by the addition of phen­oxy groups while maintaining π–π inter­actions between the aromatic SiPc groups.

## Chemical Context   

Organic photovoltaic (OPV) devices represent an emerging technology with immense potential for inexpensive solar energy generation. The majority of these prototypes depend on fullerenes as acceptor mol­ecules that are problematic due to their high manufacturing cost, low photovoltage generation and poor photochemical stability (Li *et al.*, 2014 ▸; Eftaiha *et al.*, 2014 ▸). Recently, examples have emerged where fullerene-free materials are being implemented into OPV devices reaching overall efficiencies of 5–7% (Li *et al.*, 2014 ▸; Eftaiha *et al.*, 2014 ▸; Cnops *et al.*, 2014 ▸; Zhang *et al.*, 2013 ▸). Among these emerging materials are the family of silicon phthalocyanines (SiPcs).

Metalphthalocyanines (MPcs) are composed of a nitro­gen-linked tetra­meric di­imino­isoindoline conjugated macrocycle that chelate a metal or metalloid through two covalent bonds and two coordination bonds (see Scheme 1). The resulting mol­ecules are highly stable materials that have been used for a variety of applications including dyes and pigments for decades. Silicon phthalocyanines (SiPcs) are characterized by having an additional two axial bonds that are perpendicular to the SiPc macrocycle. These axial groups can serve as chemical handles for the functionalization of the base SiPc mol­ecule. Such functionalizational groups can impart solubility as well as change the solid-state arrangement.
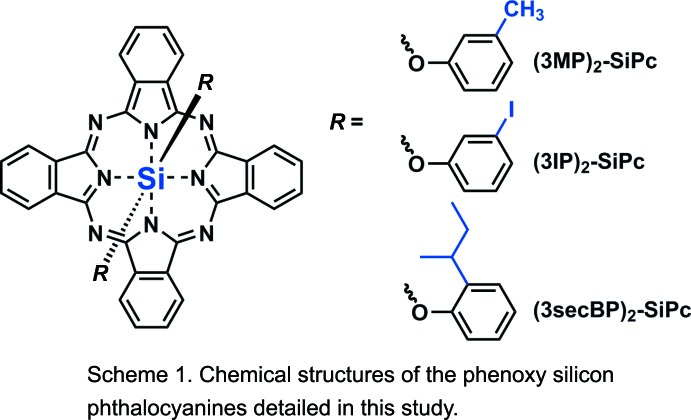


**Figure 1:**
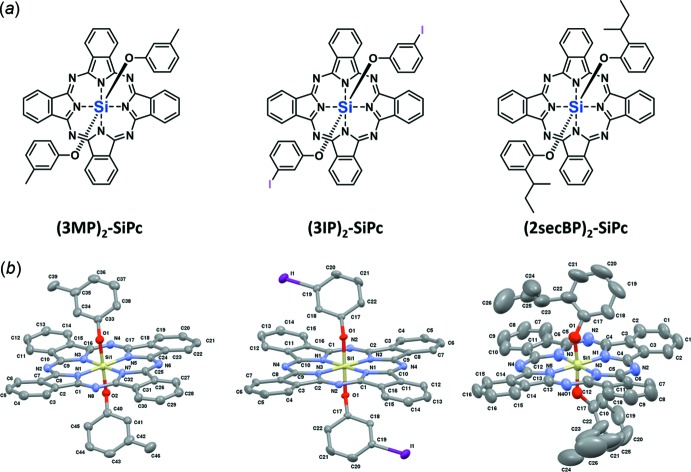
(*a*) Chemical schemes and (*b*) mol­ecular structures showing 50% probability displacement ellipsoids of (3MP)_2_-SiPc (left), (3IP)_2_-SiPc (middle) and (2*sec*BP)_2_-SiPc (right). H atoms omitted for clarity.

Honda *et al.* and our group have studied highly soluble tri-*n*-hexyl-silyl-SiPc [(3HS)_2_-SiPc] as ternary additives in bulk heterojunction (BHJ) OPV devices (Lessard *et al.*, 2014 ▸; Honda *et al.*, 2011 ▸, 2009 ▸). Our hypothesis was that the high solubility was also combined with a high tendency to crystallize into the solid state with high levels of order. As part of that study, (3HS)_2_-SiPc and an analog bis­(3-penta­decyl­phen­oxy)- SiPc [(PDP)2-SiPc] were found to have very few π–π inter­actions between the aromatic SiPc core due to the large alkyl substituents (Lessard *et al.*, 2014 ▸). Our group recently reported that simple phen­oxy­lation chemistry can be employed to enhance the π–π inter­actions present with the solid-state arrangement of the SiPc mol­ecules, resulting in improved efficiency of planar heterojunction (PHJ) OPV devices (Lessard, White *et al.*, 2015 ▸; Lessard, Grant *et al.*, 2015 ▸). Our work on boron subphthalocyanines (BsubPcs) has also illustrated that a *meta*-methyl phen­oxy group is a carbon-efficient method for significantly increasing the solubility of BsubPcs (Paton *et al.*, 2012 ▸), a characteristic that is necessary for solution-processed OPVs and other characterization techniques. In addition, 3-iodo-phen­oxy-BsubPc was found to exhibit halogen bonding between the iodo group and the BsubPc macrocycle and therefore resulting in a well-defined solid-state arrangement. The sum of these observations therefore lead our group to focus on the synthesis of soluble solution-processable phen­oxy SiPcs that may have varying degrees of carbon-efficient solubilities and tendencies to crystallize with high order into the solid state. We therefore have synthesized three new derivatives: bis­(3-methyl­phen­oxy)silicon phthalocyanine [(3MP)_2_-SiPc], bis­(2-*sec*-butyl­phen­oxy)silicon phthalo­cyanine [(2*sec*BP)_2_-SiPc] and bis­(3-iodo­phen­oxy)silicon phthalocyanine [(3IP)_2_-SiPc] (Fig. 1 ▸). We wished to investigate whether a 1- and 4-carbon solubilizing group would both enable solubility and facilitate more π–π inter­actions between the aromatic SiPc units compared to (3HS)_2_-SiPc and also to probe whether halogen bonding would be present in crystals grown of (3IP)_2_-SiPc (Virdo *et al.*, 2013 ▸).

**Figure 2:**
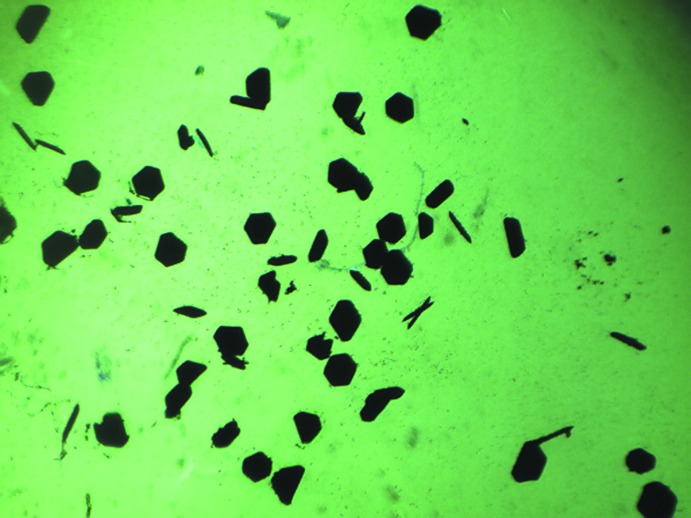
An optical microscope image of (3MP)_2_-SiPc grown by slow diffusion of heptane into THF.

Single crystals of (3MP)_2_-SiPc, (3IP)_2_-SiPc and (2*sec*BP)_2_-SiPc were grown by slow diffusion of heptane into THF and were characterized by single crystal X-ray diffraction. (3MP)_2_-SiPc was also grown by slow diffusion of pentane into benzene and evaporation form chloro­form, resulting in identical crystals as identified by X-ray crystallography. Fig. 2 ▸ is a picture of actual crystals of (3MP)_2_-SiPc, roughly 1.5 mm in size, grown by slow evaporation.

## Structural commentary   

Of note at the structural level, when considering the three reported structures, is the relatively higher disorder observed for (2*sec*BP)_2_-SiPc in the solid state (as indicted by the size of the ellipsoids, Fig. 1 ▸) compared to that of (3MP)_2_-SiPc, (3IP)_2_-SiPc and other known bis-phen­oxy-SiPc structures (Lessard, Grant *et al.*, 2015 ▸). This is consistent with the very high solubility observed for (2*sec*BP)_2_-SiPc and in contrast to the low disorder observed for the also highly soluble (3HS)_2_-SiPc) (Lessard *et al.*, 2014 ▸).

## Supermolecular Features   

**Figure 3:**
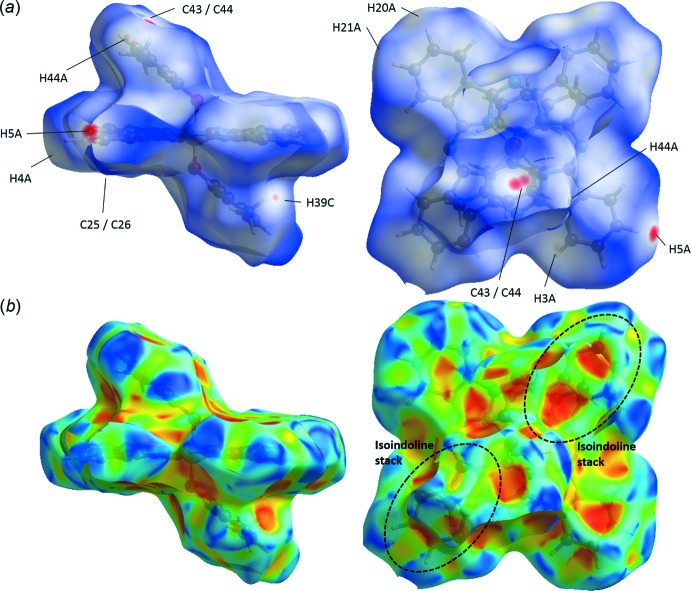
Hirshfeld surface analysis of (3MP)_2_-SiPc mapped with (*a*) *d_norm_* and (*b*) shape index. Red spots on the *d_norm_* surface indicate contacts at distances closer than the sum of the corresponding van der Waals radii. Significant π–π inter­actions between (3MP)_2_-SiPc are outlined by the dashed black circle.

**Figure 4:**
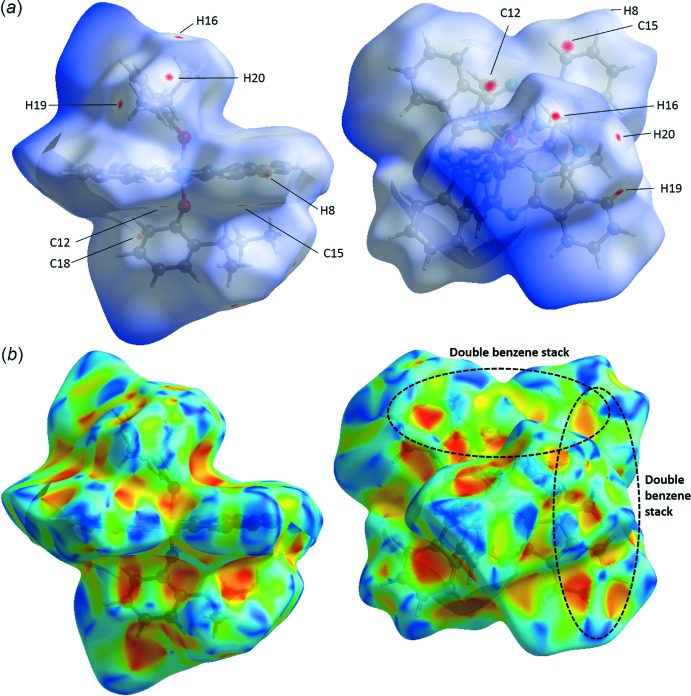
Hirshfeld surface analysis of (2*sec*BP)_2_-SiPc mapped with (*a*) *d_norm_* and (*b*) shape index. Red spots on the *d_norm_* surface indicate contacts at distances closer than the sum of the corresponding van der Waals radii. Significant π–π inter­actions between (2*sec*BP)_2_-SiPc are outlined by the dashed black circle.

**Figure 5:**
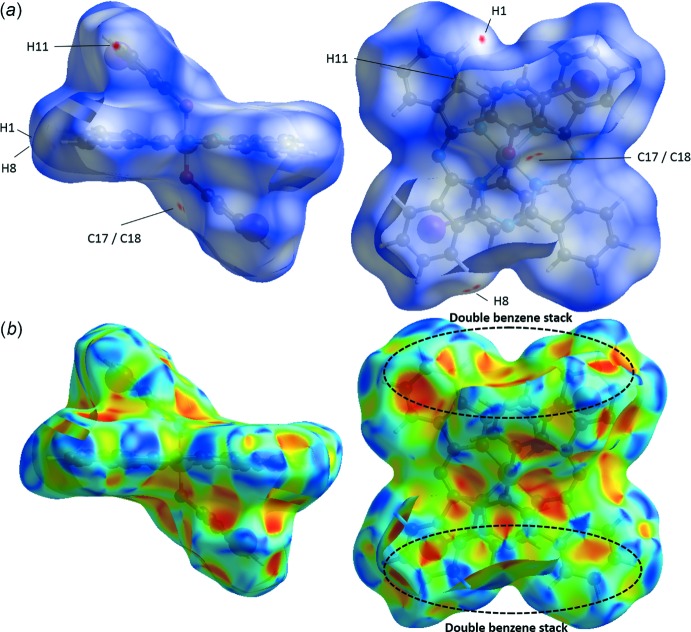
Hirshfeld surface analysis of (3IP)_2_-SiPc mapped with (*a*) *d_norm_* and (*b*) shape index. Red spots on the *d_norm_* surface indicate contacts at distances closer than the sum of the corresponding van der Waals radii. Significant π–π inter­actions between (3IP)_2_-SiPc are outlined by the dashed black circle.

The crystal structures were studied using Hirshfeld surface (HS) analysis (Spackman & Jayatilaka, 2009 ▸). All three crystals were mapped using (*a*) *d_norm_* and (*b*) shape index in Fig. 3 ▸ for (3MP)_2_-SiPc, Fig. 4 ▸ for (3IP)_2_-SiPc and Fig. 5 ▸ for (2*sec*BP)_2_-SiPc. In all three figures, the regions shaded in red correspond to the contacts at distances shorter than the sum of the van der Waals radii while the white to blue are for the distances longer than the sum of the van der Waals radii. In each crystal, the close contacts (and their symmetry equivalents) are readily identified on these maps and in all three cases they are different. For example for (3MP)_2_-SiPc (Fig. 3 ▸) one of the hydrogen atoms (H39*C*) of the 3-methyl group on the phen­oxy group experiences a contact of a distance of 2.341 Å (C39—H39*C* **⋯**H3*A*—C3; Table 1 ▸). It is inter­esting to note that for (3IP)_2_-SiPc, the iodo group does not have any significant inter­actions with adjacent mol­ecules (Fig. 2 ▸ *a*). These observations are not consistent with our previous observations for various halo-phen­oxy-BsubPcs such as 3-iodo-phen­oxy BsubPc (Virdo *et al.*, 2013 ▸). The shape index (Fig. 3 ▸ *b*, 4*b*, 5*b*) is based on the two local principal curvatures of the HS, with concave regions shaded in red and convex regions shaded in blue (Spackman & Jayatilaka, 2009 ▸). Again, these plots illustrate the difference in the solid-state arrangement between all three mol­ecules (Fig. 3 ▸ *b*, 4*b*, 5*b*). Unfortunately, similarly to previously reported carbazole derivatives (Rozycka-Sokolowska *et al.*, 2015 ▸), these plots do not generate further insight into the π–π inter­actions between mol­ecules due to their relatively large distances of 3.5–4.0 Å.

**Figure 6:**
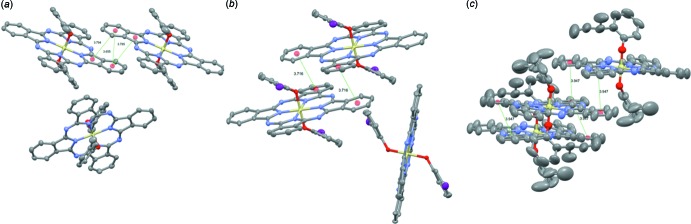
Part of the crystal structure of (*a*) (3MP)_2_-SiPc, (*b*) (3IP)_2_-SiPc and (*c*) (2secBP)_2_-SiPc. The dotted green lines represent significant π–π inter­actions with a centroid–centroid distance < 4.0 Å. Details on the π-π inter­actions are tabulated in Table 3 ▸.

Being inter­ested in the stacking between aromatic macrocycles, we previously established (Lessard, Grant *et al.*, 2015 ▸) criteria to compare the π–π inter­actions between neighboring Pc mol­ecules for single crystals of SiPcs. Following these established criteria, the π–π inter­actions of (3MP)_2_-SiPc were identified and compared to previously published phen­oxy SiPcs (Table 2 ▸). Fig. 6 ▸ *a* illustrates the packing of (3MP)_2_-SiPc crystals which is very similar to the packing of previously reported bis­(3,4,5-tri­fluoro­phen­oxy) SiPc [(345F)_2_-SiPc; Lessard, Grant *et al.*, 2015 ▸]. For example, both mol­ecules experience a complete isoindoline stacking where the shortest mol­ecular distances between isoindoline groups of (3MP)_2_-SiPc and (345FP)_2_-SiPc were determined to be 3.655 and 3.580 Å, respectively. In addition, the (3MP)_2_-SiPc exhibits a slip angle of 22.33/22.53° with a slight offset of 0.21° between the aromatic planes while (345F)_2_-SiPc has a less significant slip angle of 18.90° and exactly parallel (0° between planes) inter­acting isoindoline groups (Fig. 6 ▸ *b*).

These results indicate that (3MP)_2_-SiPc has similar inter­actions to (345F)_2_-SiPc, which represents significant increases in π–π inter­action between SiPc groups compared to the starting Cl_2_-SiPc mol­ecule. (3IP)_2_-SiPc and (2*sec*BP)_2_-SiPc on the other hand exhibit a parallel stacking of two of the peripheral aromatic groups. Of the SiPcs similar to (35F)_2_-SiPc and (246F)_2_-SiPc (Lessard, White *et al.*, 2015 ▸; Lessard, Grant *et al.*, 2015 ▸), for example, (3IP)_2_-SiPc experienced a similar stacking to (246F)_2_-SiPc (Lessard, Grant *et al.*, 2015 ▸), both having a parallel stacking of two of the peripheral aromatic units of the SiPc chromophore, with very similar inter-ring distances of 3.716 and 3.860 Å, respectively, suggesting similar strength in π–π inter­actions between neighboring mol­ecules for both (3IP)_2_-SiPc and (246F)_2_-SiPc (Fig. 6 ▸, Table 2 ▸). (3IP)_2_-SiPc has a slip angle of 17.55/14.60° with 10.99° between the aromatic planes while (246F)_2_-SiPc has a more significant slip angle of 30.08° and completely parallel (0° between planes) and inter­acting aromatic groups (Fig. 6 ▸, Table 2 ▸). (2*sec*BP)_2_-SiPc has a unique two-dimensional stacking where two peripheral aromatic groups will stack with an adjacent SiPc mol­ecule and one of the same peripheral aromatic groups along with a third one will stack in a similar fashion but at 90° from the first inter­action (Fig. 6 ▸ *c*, Table 2 ▸). In both cases a relatively large inter-ring distance of 3.947 Å was observed, suggesting a weak π-π inter­actions between neighboring (2*sec*BP)_2_-SiPcs (Fig. 6 ▸, Table 2 ▸). This weak inter­action is not a surprise due to the additional solubilizing groups (*sec*-but­yl) which space out the mol­ecules and increase the size of the unit cell.

## Synthesis and crystallization   

**Materials**

*m*-Cresol (>98%) 2-*sec*-butyl­phenol (98%) and 3-iodo­phenol (98%) were obtained from Sigma–Aldrich and chloro­benzene (99.5%) and chloro­form (CHCl_3_, 99.8%) were obtained from Caledon Laboratories Ltd. All chemicals were used as received unless otherwise specified. Di­chloro silicon phthalocyanine (Cl_2_-SiPc) was synthesized according to the literature (Lowery *et al.* 1965 ▸).

**Synthesis of silicon phthalocyanine derivatives**

The synthesis of (3MP)_2_-SiPc, (3IP)_2_-SiPcs and (2*sec*BP)_2_-SiPcs were performed following the general procedure used to synthesize F_10_-SiPc·(Lessard, White, *et al.* 2015 ▸). For example, the synthesis of (3MP)_2_-SiPc was performed in a round-bottom flask equipped with a condenser and nitro­gen purge, which was filled with a 10:1 molar excess of *m*-cresol (2.3g, 21 mol) to Cl_2_-SiPc (1.3g, 2.1 mol) in chloro­benzene (100 ml). The mixture was stirred and heated to 388 K overnight and cooled to room temperature. The product was then obtained by precipitation into iso­propanol and filtered. The product was then dried in a vacuum oven overnight. Yield: 1.3g (80.2 mol%). DART Mass spectroscopy: calculated mass: 755.234, obtained mass: 755.236. (3IP)_2_-SiPcs and (2*sec*BP)_2_-SiPcs were synthesized under similar conditions and crystals were again obtained by slow diffusion of heptane into a THF solution.

## Refinement   

Crystal data collection and structure refinement details are summarized in Table 3 ▸. H atoms were placed in calculated positions C—H = 0.94–0.98 Å and included in a riding-motion approximation with *U*_iso_(H) = 1.2*U*_eq_(C) or 1.5*U*_eq_(C_meth­yl_).

In (3MP)_2_-Si there appears to be pseudosymmetry with an approximate centre of symmetry. The *c*-glide reflections are weak but present and the *P*2_1_/*c* structure refines only to *ca R*1 = 10% compared to 4.4% for the *P*2_1_ structure. The crystal is an inversion twin with a ratio of components of 0.51 (4):0.49 (4).

During the refinement of (2*sec*BP)_2_-SiPc, electron density peaks were located that were believed to be highly disordered solvent mol­ecules (possibly penta­ne/di­chloro­methane). Attempts made to model the solvent mol­ecule were not successful. The SQUEEZE option (Spek, 2015 ▸) in *PLATON* (Spek, 2009 ▸) indicated there was a large solvent cavity 367 A^3^. In the final cycles of refinement, this contribution (99 electrons) to the electron density was removed from the observed data. The density, the *F*(000) value, the mol­ecular weight and the formula are given without taking into account the results obtained with SQUEEZE. Similar treatments of disordered solvent mol­ecules were carried out by Stähler *et al.* (2001 ▸), Cox *et al.* (2003 ▸), Mohamed *et al.* (2003 ▸) and Athimoolam *et al.* (2005 ▸).

The crystal of (2*sec*BP)_2_-SiPc was a non-merehedral twin with a twin law determined by *CELL_NOW* (Bruker, 2011 ▸) of 0.1 0.0 0.0, 0.1 1.0 0.0, 0.3 0.0 1.0. The data were detwinned using *TWINABS* (Bruker, 2011 ▸) giving twin fractions in the ratio 0.92:0.08.

## Supplementary Material

Crystal structure: contains datablock(s) 3MP2-SiPc, 3IP2-SiPc, 2secBP2-SiPc. DOI: 10.1107/S205698901600935X/hb7581sup1.cif

Structure factors: contains datablock(s) 3MP2-SiPc. DOI: 10.1107/S205698901600935X/hb75813MP2-SiPcsup3.hkl

Structure factors: contains datablock(s) 3IP2-SiPc. DOI: 10.1107/S205698901600935X/hb75813IP2-SiPcsup2.hkl

Structure factors: contains datablock(s) 2secBP2-SiPc. DOI: 10.1107/S205698901600935X/hb75812secBP2-SiPcsup4.hkl

Supporting information file. DOI: 10.1107/S205698901600935X/hb7581sup5.pdf

Supporting information file. DOI: 10.1107/S205698901600935X/hb7581sup6.tif

Supporting information file. DOI: 10.1107/S205698901600935X/hb7581sup7.tif

CCDC references: 1484189, 1484188, 1484187

Additional supporting information:  crystallographic information; 3D view; checkCIF report

## Figures and Tables

**Table 1:** Comparison of contacts (Å) less than the sum of the van der Waals radii for various *meta*-functional bis­(*meta*-functional phen­oxy) silicon phthalocyanines

Mol­ecule	C(*K*)-H(*L*)—H(*M*)—C(N)	distance	*X*⋯*X*
(3MP)2-SiPc	C4—H4*A*⋯O2—Si1	2.67	H⋯O
(3MP)2-SiPc	C46—H46*B*⋯H11—C11	2.39	H⋯H
(3MP)2-SiPc	C39—H39*C*⋯H3*A*—C3	2.34	H⋯H
(3MP)2-SiPc	C42—C43⋯H21*A*—C21	2.75	C⋯H
(3IP)2-SiPc	C4—H1⋯H11—C21	2.32	H⋯H
(2*sec*BP)2-SiPc	C24—H16⋯H19—C26	2.30	H⋯H

**Table 2:** Summary of single-crystal X-ray diffraction data (Å, °) Slip angle between *Pc* aromatic = angle between centroid-to-centroid and normal of each aromatic *Pc* benzene; angle between aromatic planes = smallest angle between both planes that contain the stacking aromatic benzene rings.

Compound	details of packing	shortest distance between *Pc* aromatic	slip angle between *Pc* aromatic	angle between aromatic planes	Reference
Cl_2_—SiPc	dual benzene ring stacking	4.172, 4.172	34.87 / 36.59	1.72	Lessard, White *et al.* (2015 ▸)
(3MP)_2_-SiPc	isoindoline stacking	3.794, 3.655, 3.794	22.33 / 22.53	0.21	This work
(345F)_2_-SiPc	isoindoline stacking	3.716, 3.580, 3.716	18.90 / 18.90	0	Lessard, Grant *et al.*, (2015 ▸)
(246F)_2_-SiPc	dual benzene ring stacking	3.860, 3.860	30.08 / 30.08	0	Lessard, Grant *et al.* (2015 ▸)
(3IP)_2_-SiPc	dual benzene ring stacking	3.716, 3.716	17.55/14.60	10.9	This work
(2*sec*BP)_2_—SiPc	dual benzene ring stacking	3.947, 3.947	32.53/26.02	6.5	This work

Notes: in all cases the single crystals were grown by slow diffusion of heptane into a THF solution of the respective compound. Identical crystals of (3MP)_2_-SiPc were also grown by diffusion of pentane into a solution of benzene as well as from slow evaporation of a chloro­form solution.

**Table 3:** Experimental details

	3MP_2_-SiPc	3IP_2_-SiPc	2*sec*BP_2_-SiPc
Crystal data			
Chemical formula	C_46_H_30_N_8_O_2_Si	C_44_H_24_I_2_N_8_O_2_Si	C_52_H_42_N_8_O_2_Si
*M*_r_	754.87	978.60	839.03
Crystal system, space group	Monoclinic, *P*2_1_	Monoclinic, *P*2_1_/*c*	Orthorhombic, *I* *b* *c* *a*
Temperature (K)	147	147	220
*a*, *b*, *c* (Å)	10.2566 (4), 16.5665 (8), 11.5120 (5)	12.6431 (6), 19.587 (1), 7.5403 (4)	10.9239 (3), 25.7282 (7), 33.2065 (8)
α, β, γ (°)	90, 115.860 (3), 90	90, 103.222 (1), 90	90, 90, 90
*V* (Å^3^)	1760.20 (13)	1817.78 (16)	9332.8 (4)
*Z*	2	2	8
Radiation type	Cu *K*α	Mo *K*α	Cu *K*α
μ (mm^−1^)	1.04	1.82	0.83
Crystal size (mm)	0.27 × 0.08 × 0.03	0.40 × 0.22 × 0.04	0.12 × 0.12 × 0.01

Data collection			
Diffractometer	Bruker Kappa APEX DUO CCD	Bruker Kappa APEX DUO CCD	Bruker Kappa APEX DUO CCD
Absorption correction	Multi-scan (*SADABS*; Bruker, 2011 ▸)	Multi-scan (*SADABS*; Bruker, 2011 ▸)	Multi-scan (*TWINABS*; Bruker, 2007 ▸)
*T*_min_, *T*_max_	0.606, 0.753	0.635, 0.746	0.621, 0.753
No. of measured, independent and observed [*I* > 2σ(*I*)] reflections	11133, 5548, 4909	31089, 4119, 3721	120855, 4085, 2969
*R*_int_	0.042	0.024	0.104
(sin θ/λ)_max_ (Å^−1^)	0.595	0.650	0.596

Refinement			
*R*[*F* ^2^ > 2σ(*F* ^2^)], *wR*(*F* ^2^), *S*	0.044, 0.111, 1.03	0.037, 0.101, 1.07	0.066, 0.208, 1.08
No. of reflections	5548	4119	4085
No. of parameters	516	259	287
No. of restraints	1	0	4
H-atom treatment	H-atom parameters constrained	H-atom parameters constrained	H-atom parameters constrained
Δρ_max_, Δρ_min_ (e Å^−3^)	0.20, −0.44	2.25, −1.33	0.40, −0.36
Absolute structure	Flack (1983 ▸), 2431 Friedel pairs	–	–
Absolute structure parameter	0.51 (4)	–	–

Computer programs: *APEX2* and *SAINT* (Bruker, 2011 ▸), *SHELXS97*, *SHELXL97* and *SHELXTL* (Sheldrick, 2008 ▸), *SHELXL2013* (Sheldrick, 2015 ▸) and *Mercury* (Macrae *et al.*, 2006 ▸).

## supplementary crystallographic information

### (3MP-SiPc) Bis(3-methylphenoxy)(phthalocyanine)silicon Crystal data

**Table:**

C_46_H_30_N_8_O_2_Si	*F*(000) = 784
*M**_r_* = 754.87	*D*_x_ = 1.424 Mg m^−^^3^
Monoclinic, *P*2_1_	Cu *K*α radiation, λ = 1.54178 Å
Hall symbol: P 2yb	Cell parameters from 6829 reflections
*a* = 10.2566 (4) Å	θ = 4.3–66.2°
*b* = 16.5665 (8) Å	µ = 1.04 mm^−^^1^
*c* = 11.5120 (5) Å	*T* = 147 K
β = 115.860 (3)°	Needle, blue
*V* = 1760.20 (13) Å^3^	0.27 × 0.08 × 0.03 mm
*Z* = 2	

### (3MP-SiPc) Bis(3-methylphenoxy)(phthalocyanine)silicon Data collection

**Table:**

Bruker Kappa APEX DUO CCD diffractometer	5548 independent reflections
Radiation source: Bruker ImuS	4909 reflections with *I* > 2σ(*I*)
Multi-layer optics monochromator	*R*_int_ = 0.042
φ and ω scans	θ_max_ = 66.5°, θ_min_ = 4.3°
Absorption correction: multi-scan (SADABS; Bruker, 2011)	*h* = −11→12
*T*_min_ = 0.606, *T*_max_ = 0.753	*k* = −19→18
11133 measured reflections	*l* = −13→11

### (3MP-SiPc) Bis(3-methylphenoxy)(phthalocyanine)silicon Refinement

**Table:**

Refinement on *F*^2^	Secondary atom site location: difference Fourier map
Least-squares matrix: full	Hydrogen site location: inferred from neighbouring sites
*R*[*F*^2^ > 2σ(*F*^2^)] = 0.044	H-atom parameters constrained
*wR*(*F*^2^) = 0.111	*w* = 1/[σ^2^(*F*_o_^2^) + (0.0535*P*)^2^ + 0.6078*P*] where *P* = (*F*_o_^2^ + 2*F*_c_^2^)/3
*S* = 1.03	(Δ/σ)_max_ = 0.001
5548 reflections	Δρ_max_ = 0.20 e Å^−^^3^
516 parameters	Δρ_min_ = −0.44 e Å^−^^3^
1 restraint	Absolute structure: Flack (1983), 2431 Friedel pairs
Primary atom site location: structure-invariant direct methods	Absolute structure parameter: 0.51 (4)

### (3MP-SiPc) Bis(3-methylphenoxy)(phthalocyanine)silicon Special details

**Table:**

Geometry. All esds (except the esd in the dihedral angle between two l.s. planes) are estimated using the full covariance matrix. The cell esds are taken into account individually in the estimation of esds in distances, angles and torsion angles; correlations between esds in cell parameters are only used when they are defined by crystal symmetry. An approximate (isotropic) treatment of cell esds is used for estimating esds involving l.s. planes.
Refinement. Refinement of F^2^ against ALL reflections. The weighted R-factor wR and goodness of fit S are based on F^2^, conventional R-factors R are based on F, with F set to zero for negative F^2^. The threshold expression of F^2^ > 2sigma(F^2^) is used only for calculating R-factors(gt) etc. and is not relevant to the choice of reflections for refinement. R-factors based on F^2^ are statistically about twice as large as those based on F, and R- factors based on ALL data will be even larger.

### (3MP-SiPc) Bis(3-methylphenoxy)(phthalocyanine)silicon Fractional atomic coordinates and isotropic or equivalent isotropic displacement parameters (Å^2^)

**Table:**

	*x*	*y*	*z*	*U*_iso_*/*U*_eq_	
Si1	0.25875 (11)	0.47830 (9)	0.25868 (9)	0.02494 (17)	
O1	0.2884 (3)	0.39581 (16)	0.1824 (2)	0.0290 (6)	
O2	0.2301 (2)	0.56160 (17)	0.33650 (19)	0.0266 (6)	
N1	0.4400 (3)	0.5208 (2)	0.2753 (2)	0.0235 (7)	
N2	0.3569 (3)	0.5980 (2)	0.0763 (3)	0.0283 (7)	
N3	0.1579 (3)	0.5340 (2)	0.0995 (3)	0.0270 (7)	
N4	−0.0891 (3)	0.4903 (2)	0.0372 (2)	0.0276 (7)	
N5	0.0788 (3)	0.4350 (2)	0.2432 (2)	0.0265 (7)	
N6	0.1590 (3)	0.3578 (2)	0.4415 (3)	0.0289 (7)	
N7	0.3600 (3)	0.4220 (2)	0.4188 (2)	0.0258 (7)	
N8	0.6078 (3)	0.4650 (2)	0.4808 (3)	0.0278 (7)	
C1	0.5733 (4)	0.5069 (2)	0.3729 (3)	0.0268 (9)	
C2	0.6846 (4)	0.5463 (2)	0.3453 (3)	0.0285 (9)	
C3	0.8346 (4)	0.5475 (3)	0.4119 (3)	0.0324 (9)	
H3A	0.8835	0.5218	0.4934	0.039*	
C4	0.9092 (4)	0.5869 (3)	0.3555 (3)	0.0369 (10)	
H4A	1.0120	0.5887	0.3985	0.044*	
C5	0.8360 (4)	0.6254 (3)	0.2339 (4)	0.0346 (10)	
H5A	0.8906	0.6520	0.1966	0.042*	
C6	0.6871 (4)	0.6248 (3)	0.1691 (3)	0.0313 (9)	
H6A	0.6376	0.6513	0.0883	0.038*	
C7	0.6117 (4)	0.5839 (2)	0.2267 (3)	0.0268 (9)	
C8	0.4576 (4)	0.5688 (2)	0.1855 (3)	0.0264 (8)	
C9	0.2206 (4)	0.5802 (3)	0.0375 (3)	0.0280 (9)	
C10	0.1101 (4)	0.6066 (3)	−0.0867 (3)	0.0277 (8)	
C11	0.1158 (4)	0.6537 (3)	−0.1856 (3)	0.0341 (10)	
H11A	0.2035	0.6783	−0.1763	0.041*	
C12	−0.0104 (4)	0.6629 (3)	−0.2966 (3)	0.0346 (10)	
H12A	−0.0090	0.6938	−0.3656	0.042*	
C13	−0.1409 (4)	0.6280 (3)	−0.3111 (3)	0.0350 (10)	
H13A	−0.2258	0.6353	−0.3895	0.042*	
C14	−0.1475 (4)	0.5829 (3)	−0.2121 (3)	0.0301 (9)	
H14A	−0.2361	0.5601	−0.2205	0.036*	
C15	−0.0202 (4)	0.5725 (3)	−0.1006 (3)	0.0288 (9)	
C16	0.0115 (4)	0.5290 (3)	0.0167 (3)	0.0277 (9)	
C17	−0.0582 (4)	0.4489 (2)	0.1432 (3)	0.0267 (9)	
C18	−0.1672 (4)	0.4097 (2)	0.1710 (3)	0.0277 (9)	
C19	−0.3173 (4)	0.4088 (2)	0.1052 (3)	0.0302 (9)	
H19A	−0.3672	0.4354	0.0247	0.036*	
C20	−0.3912 (4)	0.3666 (3)	0.1636 (3)	0.0328 (9)	
H20A	−0.4939	0.3630	0.1207	0.039*	
C21	−0.3177 (4)	0.3299 (3)	0.2828 (4)	0.0352 (10)	
H21A	−0.3717	0.3032	0.3205	0.042*	
C22	−0.1689 (4)	0.3310 (3)	0.3483 (3)	0.0307 (9)	
H22A	−0.1198	0.3054	0.4299	0.037*	
C23	−0.0933 (4)	0.3709 (2)	0.2904 (3)	0.0270 (8)	
C24	0.0580 (4)	0.3869 (2)	0.3319 (3)	0.0266 (8)	
C25	0.2982 (4)	0.3746 (3)	0.4805 (3)	0.0249 (8)	
C26	0.4081 (4)	0.3485 (3)	0.6055 (3)	0.0285 (8)	
C27	0.4001 (4)	0.3019 (3)	0.7021 (3)	0.0316 (9)	
H27A	0.3114	0.2785	0.6926	0.038*	
C28	0.5274 (4)	0.2911 (3)	0.8131 (3)	0.0352 (10)	
H28A	0.5264	0.2595	0.8815	0.042*	
C29	0.6570 (4)	0.3258 (3)	0.8266 (3)	0.0334 (9)	
H29A	0.7421	0.3179	0.9047	0.040*	
C30	0.6654 (4)	0.3713 (3)	0.7299 (3)	0.0320 (9)	
H30A	0.7544	0.3939	0.7390	0.038*	
C31	0.5375 (4)	0.3825 (3)	0.6184 (3)	0.0271 (9)	
C32	0.5058 (4)	0.4273 (2)	0.5001 (3)	0.0254 (8)	
C33	0.1975 (4)	0.3625 (3)	0.0649 (3)	0.0269 (9)	
C34	0.1916 (4)	0.3949 (3)	−0.0485 (3)	0.0334 (9)	
H34A	0.2538	0.4385	−0.0443	0.040*	
C35	0.0947 (4)	0.3641 (3)	−0.1699 (3)	0.0369 (10)	
C36	0.0095 (4)	0.2991 (3)	−0.1735 (4)	0.0413 (11)	
H36A	−0.0568	0.2774	−0.2541	0.050*	
C37	0.0204 (4)	0.2654 (3)	−0.0604 (4)	0.0408 (11)	
H37A	−0.0373	0.2195	−0.0648	0.049*	
C38	0.1119 (4)	0.2957 (3)	0.0591 (3)	0.0314 (9)	
H38A	0.1165	0.2718	0.1357	0.038*	
C39	0.0801 (5)	0.4049 (3)	−0.2914 (3)	0.0517 (13)	
H39A	0.0626	0.3642	−0.3584	0.077*	
H39B	0.1695	0.4344	−0.2744	0.077*	
H39C	−0.0015	0.4428	−0.3208	0.077*	
C40	0.3230 (4)	0.5949 (3)	0.4516 (3)	0.0275 (9)	
C41	0.3322 (4)	0.5644 (3)	0.5675 (3)	0.0300 (9)	
H41A	0.2701	0.5213	0.5656	0.036*	
C42	0.4292 (4)	0.5950 (3)	0.6852 (3)	0.0375 (10)	
C43	0.5138 (4)	0.6615 (3)	0.6862 (3)	0.0377 (10)	
H43A	0.5814	0.6836	0.7659	0.045*	
C44	0.4987 (4)	0.6952 (3)	0.5704 (4)	0.0346 (9)	
H44A	0.5532	0.7419	0.5720	0.042*	
C45	0.4063 (4)	0.6623 (3)	0.4537 (3)	0.0336 (9)	
H45A	0.3991	0.6851	0.3754	0.040*	
C46	0.4508 (5)	0.5569 (3)	0.8108 (3)	0.0514 (12)	
H46A	0.4249	0.4996	0.7968	0.077*	
H46B	0.3891	0.5840	0.8440	0.077*	
H46C	0.5525	0.5622	0.8734	0.077*	

### (3MP-SiPc) Bis(3-methylphenoxy)(phthalocyanine)silicon Atomic displacement parameters (Å^2^)

**Table:**

	*U*^11^	*U*^22^	*U*^33^	*U*^12^	*U*^13^	*U*^23^
Si1	0.0259 (4)	0.0263 (4)	0.0259 (4)	−0.0001 (3)	0.0143 (3)	0.0008 (3)
O1	0.0319 (12)	0.0291 (17)	0.0296 (11)	−0.0004 (11)	0.0167 (10)	−0.0026 (10)
O2	0.0232 (11)	0.0313 (17)	0.0263 (11)	−0.0027 (10)	0.0117 (10)	−0.0046 (10)
N1	0.0230 (13)	0.0267 (18)	0.0247 (13)	−0.0033 (12)	0.0141 (12)	−0.0011 (12)
N2	0.0321 (15)	0.025 (2)	0.0295 (14)	−0.0007 (13)	0.0154 (13)	0.0011 (12)
N3	0.0293 (15)	0.028 (2)	0.0296 (13)	−0.0007 (13)	0.0181 (12)	−0.0044 (12)
N4	0.0275 (14)	0.032 (2)	0.0247 (13)	0.0007 (13)	0.0125 (12)	−0.0017 (12)
N5	0.0299 (14)	0.0259 (19)	0.0254 (13)	0.0006 (13)	0.0136 (12)	0.0001 (12)
N6	0.0329 (15)	0.029 (2)	0.0302 (14)	−0.0011 (13)	0.0186 (13)	−0.0004 (12)
N7	0.0296 (15)	0.0260 (19)	0.0264 (13)	0.0007 (13)	0.0166 (12)	0.0047 (12)
N8	0.0271 (14)	0.029 (2)	0.0320 (14)	−0.0009 (12)	0.0173 (12)	0.0016 (12)
C1	0.0330 (18)	0.027 (2)	0.0253 (16)	−0.0025 (15)	0.0177 (15)	−0.0053 (14)
C2	0.0338 (18)	0.024 (2)	0.0359 (17)	−0.0014 (16)	0.0230 (16)	−0.0017 (15)
C3	0.0306 (18)	0.036 (3)	0.0338 (17)	−0.0027 (16)	0.0167 (16)	−0.0024 (16)
C4	0.0291 (18)	0.041 (3)	0.043 (2)	−0.0025 (16)	0.0177 (17)	−0.0079 (17)
C5	0.040 (2)	0.032 (3)	0.0430 (19)	−0.0036 (17)	0.0284 (18)	0.0026 (16)
C6	0.0333 (19)	0.031 (3)	0.0369 (18)	−0.0015 (16)	0.0218 (17)	0.0027 (15)
C7	0.0313 (18)	0.025 (2)	0.0281 (16)	−0.0010 (15)	0.0162 (15)	−0.0013 (14)
C8	0.0308 (17)	0.023 (2)	0.0327 (16)	−0.0013 (15)	0.0206 (15)	−0.0071 (14)
C9	0.0333 (19)	0.026 (2)	0.0334 (17)	0.0006 (15)	0.0221 (16)	−0.0040 (15)
C10	0.0326 (18)	0.026 (2)	0.0246 (15)	0.0015 (16)	0.0120 (15)	0.0011 (15)
C11	0.0354 (19)	0.035 (3)	0.0378 (18)	−0.0009 (17)	0.0216 (17)	0.0033 (17)
C12	0.042 (2)	0.034 (3)	0.0303 (17)	0.0005 (17)	0.0177 (17)	0.0080 (16)
C13	0.040 (2)	0.035 (3)	0.0295 (17)	0.0091 (17)	0.0150 (16)	0.0040 (16)
C14	0.0299 (17)	0.026 (2)	0.0345 (17)	0.0033 (16)	0.0145 (16)	−0.0014 (15)
C15	0.0363 (19)	0.026 (2)	0.0279 (15)	0.0045 (16)	0.0173 (15)	−0.0027 (15)
C16	0.0250 (17)	0.030 (3)	0.0297 (16)	0.0028 (16)	0.0138 (15)	−0.0011 (15)
C17	0.0237 (17)	0.026 (2)	0.0297 (16)	0.0031 (15)	0.0114 (15)	−0.0018 (14)
C18	0.0281 (17)	0.027 (2)	0.0305 (16)	−0.0011 (16)	0.0155 (15)	−0.0064 (15)
C19	0.0274 (17)	0.029 (2)	0.0354 (17)	−0.0008 (15)	0.0145 (16)	−0.0080 (15)
C20	0.0295 (18)	0.033 (3)	0.0403 (19)	−0.0044 (16)	0.0195 (16)	−0.0083 (17)
C21	0.0354 (19)	0.033 (3)	0.050 (2)	−0.0074 (18)	0.0299 (18)	−0.0115 (17)
C22	0.0373 (19)	0.026 (2)	0.0344 (18)	−0.0046 (16)	0.0204 (16)	−0.0048 (15)
C23	0.0281 (18)	0.026 (2)	0.0318 (16)	−0.0011 (15)	0.0178 (15)	−0.0049 (15)
C24	0.0341 (19)	0.021 (2)	0.0288 (16)	0.0013 (16)	0.0177 (15)	0.0015 (14)
C25	0.0251 (17)	0.024 (2)	0.0275 (15)	0.0006 (14)	0.0128 (14)	0.0027 (14)
C26	0.0352 (18)	0.024 (2)	0.0326 (17)	0.0026 (16)	0.0205 (16)	0.0019 (15)
C27	0.0373 (19)	0.026 (2)	0.0326 (18)	0.0024 (16)	0.0161 (17)	0.0059 (16)
C28	0.043 (2)	0.030 (3)	0.0352 (19)	0.0047 (18)	0.0188 (17)	0.0020 (16)
C29	0.0335 (19)	0.034 (3)	0.0288 (17)	0.0014 (16)	0.0099 (16)	0.0000 (15)
C30	0.0336 (19)	0.035 (3)	0.0278 (16)	0.0002 (17)	0.0138 (15)	0.0005 (16)
C31	0.0265 (17)	0.030 (2)	0.0265 (16)	0.0019 (15)	0.0135 (15)	0.0021 (15)
C32	0.0310 (17)	0.022 (2)	0.0270 (15)	−0.0004 (15)	0.0162 (15)	−0.0009 (14)
C33	0.0258 (17)	0.028 (2)	0.0266 (16)	0.0010 (15)	0.0114 (15)	−0.0017 (15)
C34	0.0383 (19)	0.033 (3)	0.0378 (19)	0.0046 (17)	0.0245 (16)	−0.0002 (16)
C35	0.045 (2)	0.033 (3)	0.0327 (18)	0.0109 (18)	0.0172 (17)	−0.0020 (17)
C36	0.040 (2)	0.037 (3)	0.042 (2)	0.0057 (18)	0.0128 (18)	−0.0098 (18)
C37	0.038 (2)	0.033 (3)	0.051 (2)	0.0008 (17)	0.0188 (19)	−0.0056 (19)
C38	0.0345 (18)	0.024 (2)	0.044 (2)	0.0034 (16)	0.0249 (17)	0.0016 (16)
C39	0.075 (3)	0.054 (3)	0.0285 (17)	0.016 (2)	0.0245 (19)	0.0034 (18)
C40	0.0239 (16)	0.029 (2)	0.0314 (16)	0.0067 (15)	0.0138 (14)	0.0010 (15)
C41	0.0385 (19)	0.024 (2)	0.0309 (17)	−0.0026 (16)	0.0186 (16)	−0.0013 (14)
C42	0.041 (2)	0.041 (3)	0.0330 (17)	0.0140 (18)	0.0181 (16)	0.0023 (17)
C43	0.039 (2)	0.034 (3)	0.0355 (18)	0.0060 (17)	0.0115 (16)	−0.0079 (16)
C44	0.0368 (19)	0.024 (2)	0.046 (2)	−0.0021 (16)	0.0216 (18)	−0.0039 (17)
C45	0.038 (2)	0.029 (3)	0.0331 (18)	−0.0027 (17)	0.0149 (17)	−0.0001 (16)
C46	0.068 (3)	0.055 (3)	0.0336 (19)	0.013 (2)	0.024 (2)	0.0043 (19)

### (3MP-SiPc) Bis(3-methylphenoxy)(phthalocyanine)silicon Geometric parameters (Å, º)

**Table:**

Si1—O1	1.722 (3)	C18—C23	1.402 (5)
Si1—O2	1.739 (3)	C19—C20	1.401 (6)
Si1—N3	1.904 (3)	C19—H19A	0.9500
Si1—N5	1.915 (3)	C20—C21	1.384 (6)
Si1—N7	1.917 (3)	C20—H20A	0.9500
Si1—N1	1.918 (3)	C21—C22	1.377 (5)
O1—C33	1.379 (4)	C21—H21A	0.9500
O2—C40	1.367 (4)	C22—C23	1.391 (6)
N1—C1	1.358 (5)	C22—H22A	0.9500
N1—C8	1.378 (5)	C23—C24	1.435 (5)
N2—C9	1.302 (5)	C25—C26	1.453 (5)
N2—C8	1.322 (5)	C26—C27	1.386 (6)
N3—C9	1.382 (5)	C26—C31	1.389 (5)
N3—C16	1.387 (5)	C27—C28	1.384 (5)
N4—C17	1.313 (5)	C27—H27A	0.9500
N4—C16	1.320 (5)	C28—C29	1.394 (6)
N5—C24	1.383 (5)	C28—H28A	0.9500
N5—C17	1.393 (4)	C29—C30	1.377 (6)
N6—C25	1.325 (5)	C29—H29A	0.9500
N6—C24	1.326 (4)	C30—C31	1.391 (5)
N7—C32	1.378 (5)	C30—H30A	0.9500
N7—C25	1.385 (5)	C31—C32	1.457 (5)
N8—C32	1.318 (5)	C33—C34	1.389 (5)
N8—C1	1.329 (5)	C33—C38	1.395 (6)
C1—C2	1.464 (5)	C34—C35	1.410 (5)
C2—C7	1.386 (5)	C34—H34A	0.9500
C2—C3	1.388 (5)	C35—C36	1.377 (7)
C3—C4	1.367 (6)	C35—C39	1.501 (6)
C3—H3A	0.9500	C36—C37	1.376 (7)
C4—C5	1.418 (6)	C36—H36A	0.9500
C4—H4A	0.9500	C37—C38	1.378 (5)
C5—C6	1.377 (5)	C37—H37A	0.9500
C5—H5A	0.9500	C38—H38A	0.9500
C6—C7	1.395 (5)	C39—H39A	0.9800
C6—H6A	0.9500	C39—H39B	0.9800
C7—C8	1.459 (5)	C39—H39C	0.9800
C9—C10	1.451 (5)	C40—C41	1.391 (5)
C10—C15	1.395 (6)	C40—C45	1.400 (6)
C10—C11	1.402 (6)	C41—C42	1.381 (5)
C11—C12	1.374 (5)	C41—H41A	0.9500
C11—H11A	0.9500	C42—C43	1.399 (7)
C12—C13	1.398 (6)	C42—C46	1.503 (6)
C12—H12A	0.9500	C43—C44	1.390 (6)
C13—C14	1.390 (6)	C43—H43A	0.9500
C13—H13A	0.9500	C44—C45	1.374 (5)
C14—C15	1.385 (5)	C44—H44A	0.9500
C14—H14A	0.9500	C45—H45A	0.9500
C15—C16	1.437 (5)	C46—H46A	0.9800
C17—C18	1.444 (6)	C46—H46B	0.9800
C18—C19	1.387 (5)	C46—H46C	0.9800
			
O1—Si1—O2	179.59 (14)	C20—C19—H19A	121.7
O1—Si1—N3	92.10 (13)	C21—C20—C19	121.3 (3)
O2—Si1—N3	88.17 (13)	C21—C20—H20A	119.4
O1—Si1—N5	91.88 (14)	C19—C20—H20A	119.4
O2—Si1—N5	88.43 (13)	C22—C21—C20	122.2 (4)
N3—Si1—N5	89.72 (13)	C22—C21—H21A	118.9
O1—Si1—N7	87.84 (13)	C20—C21—H21A	118.9
O2—Si1—N7	91.89 (13)	C21—C22—C23	117.4 (4)
N3—Si1—N7	179.9 (2)	C21—C22—H22A	121.3
N5—Si1—N7	90.18 (13)	C23—C22—H22A	121.3
O1—Si1—N1	87.88 (13)	C22—C23—C18	120.7 (3)
O2—Si1—N1	91.81 (14)	C22—C23—C24	132.5 (3)
N3—Si1—N1	90.80 (13)	C18—C23—C24	106.6 (3)
N5—Si1—N1	179.44 (19)	N6—C24—N5	127.2 (3)
N7—Si1—N1	89.30 (13)	N6—C24—C23	122.2 (3)
C33—O1—Si1	128.6 (2)	N5—C24—C23	110.6 (3)
C40—O2—Si1	128.2 (2)	N6—C25—N7	127.7 (3)
C1—N1—C8	107.7 (3)	N6—C25—C26	122.0 (3)
C1—N1—Si1	127.1 (3)	N7—C25—C26	110.2 (3)
C8—N1—Si1	125.2 (2)	C27—C26—C31	121.9 (3)
C9—N2—C8	121.4 (3)	C27—C26—C25	131.9 (3)
C9—N3—C16	106.6 (3)	C31—C26—C25	106.2 (3)
C9—N3—Si1	125.9 (2)	C28—C27—C26	116.8 (4)
C16—N3—Si1	127.1 (3)	C28—C27—H27A	121.6
C17—N4—C16	122.0 (3)	C26—C27—H27A	121.6
C24—N5—C17	106.3 (3)	C27—C28—C29	121.3 (4)
C24—N5—Si1	126.8 (2)	C27—C28—H28A	119.3
C17—N5—Si1	126.8 (3)	C29—C28—H28A	119.3
C25—N6—C24	121.7 (3)	C30—C29—C28	121.9 (3)
C32—N7—C25	106.9 (3)	C30—C29—H29A	119.1
C32—N7—Si1	126.5 (3)	C28—C29—H29A	119.1
C25—N7—Si1	126.3 (2)	C29—C30—C31	116.9 (4)
C32—N8—C1	119.9 (3)	C29—C30—H30A	121.5
N8—C1—N1	128.5 (4)	C31—C30—H30A	121.5
N8—C1—C2	121.4 (3)	C26—C31—C30	121.1 (3)
N1—C1—C2	110.1 (3)	C26—C31—C32	107.0 (3)
C7—C2—C3	121.9 (4)	C30—C31—C32	131.8 (4)
C7—C2—C1	106.1 (3)	N8—C32—N7	128.4 (3)
C3—C2—C1	131.9 (3)	N8—C32—C31	121.9 (3)
C4—C3—C2	117.4 (3)	N7—C32—C31	109.7 (3)
C4—C3—H3A	121.3	O1—C33—C34	120.0 (4)
C2—C3—H3A	121.3	O1—C33—C38	120.4 (3)
C3—C4—C5	121.2 (3)	C34—C33—C38	119.5 (3)
C3—C4—H4A	119.4	C33—C34—C35	121.0 (4)
C5—C4—H4A	119.4	C33—C34—H34A	119.5
C6—C5—C4	121.1 (4)	C35—C34—H34A	119.5
C6—C5—H5A	119.5	C36—C35—C34	118.5 (4)
C4—C5—H5A	119.5	C36—C35—C39	121.3 (4)
C5—C6—C7	117.4 (3)	C34—C35—C39	120.1 (4)
C5—C6—H6A	121.3	C37—C36—C35	120.1 (4)
C7—C6—H6A	121.3	C37—C36—H36A	120.0
C2—C7—C6	120.9 (3)	C35—C36—H36A	120.0
C2—C7—C8	106.6 (3)	C36—C37—C38	122.3 (4)
C6—C7—C8	132.4 (3)	C36—C37—H37A	118.8
N2—C8—N1	128.4 (3)	C38—C37—H37A	118.8
N2—C8—C7	122.2 (3)	C37—C38—C33	118.6 (4)
N1—C8—C7	109.4 (3)	C37—C38—H38A	120.7
N2—C9—N3	128.3 (3)	C33—C38—H38A	120.7
N2—C9—C10	122.0 (4)	C35—C39—H39A	109.5
N3—C9—C10	109.7 (3)	C35—C39—H39B	109.5
C15—C10—C11	120.6 (3)	H39A—C39—H39B	109.5
C15—C10—C9	106.8 (3)	C35—C39—H39C	109.5
C11—C10—C9	132.6 (3)	H39A—C39—H39C	109.5
C12—C11—C10	117.4 (4)	H39B—C39—H39C	109.5
C12—C11—H11A	121.3	O2—C40—C41	120.7 (4)
C10—C11—H11A	121.3	O2—C40—C45	120.1 (3)
C11—C12—C13	122.0 (4)	C41—C40—C45	119.2 (3)
C11—C12—H12A	119.0	C42—C41—C40	121.7 (4)
C13—C12—H12A	119.0	C42—C41—H41A	119.2
C14—C13—C12	120.7 (3)	C40—C41—H41A	119.2
C14—C13—H13A	119.6	C41—C42—C43	118.5 (4)
C12—C13—H13A	119.6	C41—C42—C46	122.0 (4)
C15—C14—C13	117.6 (4)	C43—C42—C46	119.5 (4)
C15—C14—H14A	121.2	C44—C43—C42	119.9 (3)
C13—C14—H14A	121.2	C44—C43—H43A	120.0
C14—C15—C10	121.6 (4)	C42—C43—H43A	120.0
C14—C15—C16	131.9 (4)	C45—C44—C43	121.3 (4)
C10—C15—C16	106.4 (3)	C45—C44—H44A	119.4
N4—C16—N3	127.1 (3)	C43—C44—H44A	119.4
N4—C16—C15	122.5 (3)	C44—C45—C40	119.2 (4)
N3—C16—C15	110.4 (3)	C44—C45—H45A	120.4
N4—C17—N5	127.0 (4)	C40—C45—H45A	120.4
N4—C17—C18	123.0 (3)	C42—C46—H46A	109.5
N5—C17—C18	110.0 (3)	C42—C46—H46B	109.5
C19—C18—C23	121.8 (4)	H46A—C46—H46B	109.5
C19—C18—C17	131.7 (4)	C42—C46—H46C	109.5
C23—C18—C17	106.4 (3)	H46A—C46—H46C	109.5
C18—C19—C20	116.6 (4)	H46B—C46—H46C	109.5
C18—C19—H19A	121.7		

### (3IP2-SiPc) Bis(2-sec-butylphenoxy)(phthalocyanine)silicon Crystal data

**Table:**

C_44_H_24_I_2_N_8_O_2_Si	*F*(000) = 960
*M**_r_* = 978.60	*D*_x_ = 1.788 Mg m^−^^3^
Monoclinic, *P*2_1_/*c*	Mo *K*α radiation, λ = 0.71073 Å
*a* = 12.6431 (6) Å	Cell parameters from 9148 reflections
*b* = 19.587 (1) Å	θ = 2.7–27.5°
*c* = 7.5403 (4) Å	µ = 1.82 mm^−^^1^
β = 103.222 (1)°	*T* = 147 K
*V* = 1817.78 (16) Å^3^	Plate, blue
*Z* = 2	0.40 × 0.22 × 0.04 mm

### (3IP2-SiPc) Bis(2-sec-butylphenoxy)(phthalocyanine)silicon Data collection

**Table:**

Bruker Kappa APEX DUO CCD diffractometer	3721 reflections with *I* > 2σ(*I*)
Radiation source: sealed tube with Bruker Triumph monocnromator	*R*_int_ = 0.024
φ and ω scans	θ_max_ = 27.5°, θ_min_ = 1.7°
Absorption correction: multi-scan (SADABS; Bruker, 2011)	*h* = −16→16
*T*_min_ = 0.635, *T*_max_ = 0.746	*k* = −25→25
31089 measured reflections	*l* = −9→7
4119 independent reflections	

### (3IP2-SiPc) Bis(2-sec-butylphenoxy)(phthalocyanine)silicon Refinement

**Table:**

Refinement on *F*^2^	0 restraints
Least-squares matrix: full	Hydrogen site location: inferred from neighbouring sites
*R*[*F*^2^ > 2σ(*F*^2^)] = 0.037	H-atom parameters constrained
*wR*(*F*^2^) = 0.101	*w* = 1/[σ^2^(*F*_o_^2^) + (0.0505*P*)^2^ + 4.7192*P*] where *P* = (*F*_o_^2^ + 2*F*_c_^2^)/3
*S* = 1.07	(Δ/σ)_max_ < 0.001
4119 reflections	Δρ_max_ = 2.25 e Å^−^^3^
259 parameters	Δρ_min_ = −1.33 e Å^−^^3^

### (3IP2-SiPc) Bis(2-sec-butylphenoxy)(phthalocyanine)silicon Special details

**Table:**

Geometry. All esds (except the esd in the dihedral angle between two l.s. planes) are estimated using the full covariance matrix. The cell esds are taken into account individually in the estimation of esds in distances, angles and torsion angles; correlations between esds in cell parameters are only used when they are defined by crystal symmetry. An approximate (isotropic) treatment of cell esds is used for estimating esds involving l.s. planes.

### (3IP2-SiPc) Bis(2-sec-butylphenoxy)(phthalocyanine)silicon Fractional atomic coordinates and isotropic or equivalent isotropic displacement parameters (Å^2^)

**Table:**

	*x*	*y*	*z*	*U*_iso_*/*U*_eq_	
I1	0.58373 (2)	0.18313 (2)	0.00675 (4)	0.03731 (10)	
Si1	1.0000	0.0000	0.0000	0.0150 (2)	
O1	0.95195 (16)	0.07333 (10)	−0.1198 (3)	0.0179 (4)	
N1	0.88930 (19)	0.00288 (12)	0.1341 (3)	0.0169 (4)	
N2	0.9741 (2)	0.07375 (12)	0.3918 (3)	0.0193 (5)	
N3	1.09243 (18)	0.05331 (12)	0.1863 (3)	0.0165 (4)	
N4	1.25478 (19)	0.06065 (12)	0.0671 (3)	0.0195 (5)	
C1	0.8921 (2)	0.03894 (14)	0.2930 (4)	0.0176 (5)	
C2	1.0663 (2)	0.07947 (14)	0.3410 (4)	0.0179 (5)	
C3	1.1573 (2)	0.11803 (14)	0.4453 (4)	0.0201 (5)	
C4	1.1716 (3)	0.15271 (16)	0.6108 (4)	0.0254 (6)	
H4A	1.1165	0.1537	0.6778	0.031*	
C5	1.2702 (3)	0.18575 (16)	0.6727 (5)	0.0298 (7)	
H5A	1.2828	0.2102	0.7843	0.036*	
C6	1.3518 (3)	0.18382 (16)	0.5743 (5)	0.0293 (7)	
H6A	1.4180	0.2074	0.6208	0.035*	
C7	1.3387 (3)	0.14851 (15)	0.4112 (4)	0.0248 (6)	
H7A	1.3945	0.1468	0.3455	0.030*	
C8	1.2387 (2)	0.11562 (14)	0.3486 (4)	0.0196 (5)	
C9	1.1969 (2)	0.07466 (14)	0.1873 (4)	0.0180 (5)	
C10	1.2146 (2)	0.02365 (14)	−0.0789 (4)	0.0179 (5)	
C11	1.2804 (2)	0.00286 (14)	−0.2035 (4)	0.0199 (5)	
C12	1.3895 (2)	0.01224 (16)	−0.2023 (4)	0.0238 (6)	
H12A	1.4349	0.0400	−0.1130	0.029*	
C13	1.4293 (3)	−0.02018 (18)	−0.3354 (5)	0.0289 (7)	
H13A	1.5042	−0.0163	−0.3348	0.035*	
C14	1.3613 (3)	−0.05895 (17)	−0.4722 (4)	0.0280 (6)	
H14A	1.3911	−0.0802	−0.5630	0.034*	
C15	1.2519 (3)	−0.06679 (15)	−0.4774 (4)	0.0227 (6)	
H15A	1.2054	−0.0918	−0.5719	0.027*	
C16	1.2129 (2)	−0.03648 (14)	−0.3382 (4)	0.0193 (5)	
C17	0.9045 (2)	0.12947 (14)	−0.0640 (4)	0.0180 (5)	
C18	0.7937 (2)	0.12940 (14)	−0.0651 (4)	0.0192 (5)	
H18A	0.7506	0.0901	−0.1044	0.023*	
C19	0.7474 (2)	0.18679 (15)	−0.0088 (4)	0.0223 (6)	
C20	0.8061 (3)	0.24578 (17)	0.0427 (5)	0.0317 (7)	
H20A	0.7728	0.2848	0.0816	0.038*	
C21	0.9147 (3)	0.24686 (17)	0.0365 (5)	0.0342 (7)	
H21A	0.9555	0.2876	0.0667	0.041*	
C22	0.9651 (3)	0.18879 (15)	−0.0136 (4)	0.0246 (6)	
H22A	1.0402	0.1897	−0.0134	0.030*	

### (3IP2-SiPc) Bis(2-sec-butylphenoxy)(phthalocyanine)silicon Atomic displacement parameters (Å^2^)

**Table:**

	*U*^11^	*U*^22^	*U*^33^	*U*^12^	*U*^13^	*U*^23^
I1	0.02480 (14)	0.04162 (16)	0.04785 (17)	0.00971 (8)	0.01317 (10)	0.00607 (10)
Si1	0.0145 (5)	0.0154 (5)	0.0134 (5)	0.0021 (4)	−0.0001 (4)	−0.0025 (4)
O1	0.0204 (9)	0.0172 (9)	0.0149 (9)	0.0057 (7)	0.0016 (7)	−0.0005 (7)
N1	0.0174 (11)	0.0157 (11)	0.0159 (10)	0.0014 (8)	0.0000 (8)	−0.0015 (8)
N2	0.0215 (12)	0.0198 (11)	0.0155 (11)	0.0030 (9)	0.0016 (9)	−0.0022 (8)
N3	0.0158 (10)	0.0175 (11)	0.0145 (10)	0.0018 (8)	0.0000 (8)	−0.0026 (8)
N4	0.0190 (11)	0.0189 (11)	0.0196 (11)	−0.0014 (9)	0.0021 (9)	−0.0002 (9)
C1	0.0211 (13)	0.0153 (12)	0.0157 (12)	0.0043 (10)	0.0029 (10)	0.0002 (9)
C2	0.0209 (13)	0.0153 (12)	0.0149 (12)	0.0025 (10)	−0.0014 (10)	−0.0026 (9)
C3	0.0229 (14)	0.0169 (13)	0.0170 (13)	0.0025 (10)	−0.0025 (10)	−0.0008 (10)
C4	0.0295 (15)	0.0222 (14)	0.0205 (14)	0.0033 (12)	−0.0026 (11)	−0.0052 (11)
C5	0.0363 (18)	0.0219 (15)	0.0243 (15)	−0.0023 (12)	−0.0074 (13)	−0.0073 (11)
C6	0.0322 (17)	0.0227 (15)	0.0266 (16)	−0.0081 (12)	−0.0065 (13)	−0.0014 (11)
C7	0.0247 (15)	0.0194 (14)	0.0261 (15)	−0.0028 (11)	−0.0027 (11)	0.0008 (11)
C8	0.0218 (13)	0.0153 (12)	0.0180 (13)	0.0010 (10)	−0.0031 (10)	−0.0012 (10)
C9	0.0165 (12)	0.0166 (12)	0.0180 (13)	0.0004 (10)	−0.0024 (10)	−0.0012 (10)
C10	0.0185 (13)	0.0162 (12)	0.0182 (12)	0.0010 (10)	0.0028 (10)	0.0016 (10)
C11	0.0231 (14)	0.0175 (13)	0.0189 (13)	0.0009 (10)	0.0043 (11)	0.0027 (10)
C12	0.0231 (14)	0.0264 (15)	0.0219 (14)	−0.0049 (11)	0.0053 (11)	0.0017 (11)
C13	0.0243 (15)	0.0351 (17)	0.0305 (16)	−0.0032 (13)	0.0128 (12)	0.0025 (13)
C14	0.0333 (17)	0.0305 (16)	0.0246 (15)	0.0005 (13)	0.0159 (13)	−0.0009 (12)
C15	0.0280 (15)	0.0212 (14)	0.0199 (13)	−0.0016 (11)	0.0078 (11)	−0.0007 (10)
C16	0.0222 (13)	0.0170 (13)	0.0183 (13)	0.0009 (10)	0.0040 (10)	0.0025 (10)
C17	0.0222 (13)	0.0165 (12)	0.0144 (12)	0.0024 (10)	0.0021 (10)	−0.0007 (9)
C18	0.0217 (13)	0.0182 (13)	0.0159 (12)	−0.0006 (10)	0.0008 (10)	0.0027 (10)
C19	0.0200 (14)	0.0256 (15)	0.0217 (14)	0.0035 (11)	0.0054 (11)	0.0039 (10)
C20	0.0393 (19)	0.0215 (15)	0.0365 (18)	0.0048 (13)	0.0132 (14)	−0.0069 (13)
C21	0.0388 (19)	0.0211 (15)	0.0426 (19)	−0.0084 (13)	0.0092 (15)	−0.0101 (13)
C22	0.0216 (15)	0.0233 (15)	0.0282 (16)	−0.0027 (11)	0.0041 (12)	−0.0017 (11)

### (3IP2-SiPc) Bis(2-sec-butylphenoxy)(phthalocyanine)silicon Geometric parameters (Å, º)

**Table:**

I1—C19	2.100 (3)	C7—C8	1.401 (4)
Si1—O1^i^	1.7314 (19)	C7—H7A	0.9500
Si1—O1	1.7314 (19)	C8—C9	1.452 (4)
Si1—N1	1.906 (2)	C10—N1^i^	1.385 (4)
Si1—N1^i^	1.906 (2)	C10—C11	1.449 (4)
Si1—N3^i^	1.918 (2)	C11—C12	1.389 (4)
Si1—N3	1.918 (2)	C11—C16	1.399 (4)
O1—C17	1.364 (3)	C12—C13	1.376 (4)
N1—C1	1.385 (3)	C12—H12A	0.9500
N1—C10^i^	1.385 (4)	C13—C14	1.405 (5)
N2—C2	1.313 (4)	C13—H13A	0.9500
N2—C1	1.319 (4)	C14—C15	1.383 (4)
N3—C2	1.381 (3)	C14—H14A	0.9500
N3—C9	1.384 (4)	C15—C16	1.390 (4)
N4—C9	1.317 (4)	C15—H15A	0.9500
N4—C10	1.319 (4)	C16—C1^i^	1.444 (4)
C1—C16^i^	1.444 (4)	C17—C22	1.396 (4)
C2—C3	1.449 (4)	C17—C18	1.399 (4)
C3—C8	1.392 (4)	C18—C19	1.378 (4)
C3—C4	1.396 (4)	C18—H18A	0.9500
C4—C5	1.388 (5)	C19—C20	1.381 (5)
C4—H4A	0.9500	C20—C21	1.384 (5)
C5—C6	1.401 (5)	C20—H20A	0.9500
C5—H5A	0.9500	C21—C22	1.397 (5)
C6—C7	1.388 (4)	C21—H21A	0.9500
C6—H6A	0.9500	C22—H22A	0.9500
			
O1^i^—Si1—O1	180.0	C3—C8—C7	121.8 (3)
O1^i^—Si1—N1	87.72 (9)	C3—C8—C9	106.6 (2)
O1—Si1—N1	92.28 (9)	C7—C8—C9	131.6 (3)
O1^i^—Si1—N1^i^	92.28 (9)	N4—C9—N3	127.8 (2)
O1—Si1—N1^i^	87.72 (9)	N4—C9—C8	122.6 (3)
N1—Si1—N1^i^	180.0	N3—C9—C8	109.6 (2)
O1^i^—Si1—N3^i^	90.74 (9)	N4—C10—N1^i^	127.9 (3)
O1—Si1—N3^i^	89.26 (9)	N4—C10—C11	121.8 (3)
N1—Si1—N3^i^	90.37 (10)	N1^i^—C10—C11	110.2 (2)
N1^i^—Si1—N3^i^	89.63 (10)	C12—C11—C16	121.2 (3)
O1^i^—Si1—N3	89.26 (9)	C12—C11—C10	132.5 (3)
O1—Si1—N3	90.74 (9)	C16—C11—C10	106.3 (2)
N1—Si1—N3	89.63 (10)	C13—C12—C11	117.5 (3)
N1^i^—Si1—N3	90.37 (10)	C13—C12—H12A	121.2
N3^i^—Si1—N3	180.0	C11—C12—H12A	121.2
C17—O1—Si1	129.36 (17)	C12—C13—C14	121.4 (3)
C1—N1—C10^i^	106.6 (2)	C12—C13—H13A	119.3
C1—N1—Si1	126.73 (19)	C14—C13—H13A	119.3
C10^i^—N1—Si1	126.19 (18)	C15—C14—C13	121.4 (3)
C2—N2—C1	121.0 (2)	C15—C14—H14A	119.3
C2—N3—C9	107.2 (2)	C13—C14—H14A	119.3
C2—N3—Si1	126.65 (19)	C14—C15—C16	117.1 (3)
C9—N3—Si1	126.11 (19)	C14—C15—H15A	121.4
C9—N4—C10	121.4 (2)	C16—C15—H15A	121.4
N2—C1—N1	127.8 (3)	C15—C16—C11	121.3 (3)
N2—C1—C16^i^	122.0 (2)	C15—C16—C1^i^	131.9 (3)
N1—C1—C16^i^	110.1 (2)	C11—C16—C1^i^	106.7 (2)
N2—C2—N3	127.9 (2)	O1—C17—C22	120.2 (3)
N2—C2—C3	122.3 (3)	O1—C17—C18	120.5 (2)
N3—C2—C3	109.8 (2)	C22—C17—C18	119.2 (3)
C8—C3—C4	121.6 (3)	C19—C18—C17	119.5 (3)
C8—C3—C2	106.8 (2)	C19—C18—H18A	120.2
C4—C3—C2	131.6 (3)	C17—C18—H18A	120.2
C5—C4—C3	116.8 (3)	C18—C19—C20	122.0 (3)
C5—C4—H4A	121.6	C18—C19—I1	118.9 (2)
C3—C4—H4A	121.6	C20—C19—I1	119.1 (2)
C4—C5—C6	121.5 (3)	C19—C20—C21	118.5 (3)
C4—C5—H5A	119.2	C19—C20—H20A	120.7
C6—C5—H5A	119.2	C21—C20—H20A	120.7
C7—C6—C5	122.0 (3)	C20—C21—C22	120.9 (3)
C7—C6—H6A	119.0	C20—C21—H21A	119.5
C5—C6—H6A	119.0	C22—C21—H21A	119.5
C6—C7—C8	116.3 (3)	C17—C22—C21	119.7 (3)
C6—C7—H7A	121.9	C17—C22—H22A	120.1
C8—C7—H7A	121.9	C21—C22—H22A	120.1
			
N1—Si1—O1—C17	34.0 (2)	C2—N3—C9—C8	0.2 (3)
N1^i^—Si1—O1—C17	−146.0 (2)	Si1—N3—C9—C8	178.24 (18)
N3^i^—Si1—O1—C17	124.3 (2)	C3—C8—C9—N4	−178.4 (3)
N3—Si1—O1—C17	−55.7 (2)	C7—C8—C9—N4	1.3 (5)
C2—N2—C1—N1	2.3 (4)	C3—C8—C9—N3	0.4 (3)
C2—N2—C1—C16^i^	−174.9 (3)	C7—C8—C9—N3	−179.9 (3)
C10^i^—N1—C1—N2	−178.7 (3)	C9—N4—C10—N1^i^	3.0 (4)
Si1—N1—C1—N2	−6.1 (4)	C9—N4—C10—C11	−174.4 (3)
C10^i^—N1—C1—C16^i^	−1.2 (3)	N4—C10—C11—C12	2.4 (5)
Si1—N1—C1—C16^i^	171.45 (18)	N1^i^—C10—C11—C12	−175.4 (3)
C1—N2—C2—N3	1.0 (4)	N4—C10—C11—C16	178.7 (3)
C1—N2—C2—C3	179.2 (3)	N1^i^—C10—C11—C16	0.8 (3)
C9—N3—C2—N2	177.7 (3)	C16—C11—C12—C13	−1.8 (4)
Si1—N3—C2—N2	−0.4 (4)	C10—C11—C12—C13	174.0 (3)
C9—N3—C2—C3	−0.7 (3)	C11—C12—C13—C14	2.7 (5)
Si1—N3—C2—C3	−178.75 (18)	C12—C13—C14—C15	−0.8 (5)
N2—C2—C3—C8	−177.5 (3)	C13—C14—C15—C16	−2.0 (5)
N3—C2—C3—C8	1.0 (3)	C14—C15—C16—C11	2.9 (4)
N2—C2—C3—C4	2.8 (5)	C14—C15—C16—C1^i^	−174.1 (3)
N3—C2—C3—C4	−178.7 (3)	C12—C11—C16—C15	−1.0 (4)
C8—C3—C4—C5	1.2 (4)	C10—C11—C16—C15	−177.7 (3)
C2—C3—C4—C5	−179.2 (3)	C12—C11—C16—C1^i^	176.7 (3)
C3—C4—C5—C6	−0.5 (5)	C10—C11—C16—C1^i^	−0.1 (3)
C4—C5—C6—C7	−0.5 (5)	Si1—O1—C17—C22	100.8 (3)
C5—C6—C7—C8	0.9 (5)	Si1—O1—C17—C18	−82.4 (3)
C4—C3—C8—C7	−0.8 (4)	O1—C17—C18—C19	−179.8 (2)
C2—C3—C8—C7	179.5 (3)	C22—C17—C18—C19	−2.9 (4)
C4—C3—C8—C9	178.9 (3)	C17—C18—C19—C20	2.5 (4)
C2—C3—C8—C9	−0.8 (3)	C17—C18—C19—I1	−175.6 (2)
C6—C7—C8—C3	−0.3 (4)	C18—C19—C20—C21	0.2 (5)
C6—C7—C8—C9	−179.9 (3)	I1—C19—C20—C21	178.3 (3)
C10—N4—C9—N3	1.9 (4)	C19—C20—C21—C22	−2.6 (6)
C10—N4—C9—C8	−179.6 (3)	O1—C17—C22—C21	177.5 (3)
C2—N3—C9—N4	178.9 (3)	C18—C17—C22—C21	0.6 (5)
Si1—N3—C9—N4	−3.1 (4)	C20—C21—C22—C17	2.1 (5)

Symmetry code: (i) −*x*+2, −*y*, −*z*.

### (2secBP2-SiPc) Bis(3-iodophenoxy)(phthalocyanine)silicon Crystal data

**Table:**

C_52_H_42_N_8_O_2_Si	*F*(000) = 3520
*M**_r_* = 839.03	*D*_x_ = 1.194 Mg m^−^^3^
Orthorhombic, *I**b**c**a*	Cu *K*α radiation, λ = 1.54178 Å
Hall symbol: -I 2b 2c	Cell parameters from 586 reflections
*a* = 10.9239 (3) Å	θ = 4.4–35.1°
*b* = 25.7282 (7) Å	µ = 0.83 mm^−^^1^
*c* = 33.2065 (8) Å	*T* = 220 K
*V* = 9332.8 (4) Å^3^	Plate, blue
*Z* = 8	0.12 × 0.12 × 0.01 mm

### (2secBP2-SiPc) Bis(3-iodophenoxy)(phthalocyanine)silicon Data collection

**Table:**

Bruker Kappa APEX DUO CCD diffractometer	4085 independent reflections
Radiation source: fine-focus sealed tube	2969 reflections with *I* > 2σ(*I*)
Multi-layer optics monochromator	*R*_int_ = 0.104
φ and ω scans	θ_max_ = 66.8°, θ_min_ = 3.4°
Absorption correction: multi-scan (*TWINABS*; Bruker, 2007)	*h* = −12→12
*T*_min_ = 0.621, *T*_max_ = 0.753	*k* = −30→30
120855 measured reflections	*l* = −38→38

### (2secBP2-SiPc) Bis(3-iodophenoxy)(phthalocyanine)silicon Refinement

**Table:**

Refinement on *F*^2^	Primary atom site location: structure-invariant direct methods
Least-squares matrix: full	Secondary atom site location: difference Fourier map
*R*[*F*^2^ > 2σ(*F*^2^)] = 0.066	Hydrogen site location: inferred from neighbouring sites
*wR*(*F*^2^) = 0.208	H-atom parameters constrained
*S* = 1.08	*w* = 1/[σ^2^(*F*_o_^2^) + (0.1166*P*)^2^ + 6.9892*P*] where *P* = (*F*_o_^2^ + 2*F*_c_^2^)/3
4085 reflections	(Δ/σ)_max_ < 0.001
287 parameters	Δρ_max_ = 0.40 e Å^−^^3^
4 restraints	Δρ_min_ = −0.36 e Å^−^^3^

### (2secBP2-SiPc) Bis(3-iodophenoxy)(phthalocyanine)silicon Special details

**Table:**

Geometry. All esds (except the esd in the dihedral angle between two l.s. planes) are estimated using the full covariance matrix. The cell esds are taken into account individually in the estimation of esds in distances, angles and torsion angles; correlations between esds in cell parameters are only used when they are defined by crystal symmetry. An approximate (isotropic) treatment of cell esds is used for estimating esds involving l.s. planes.
Refinement. Refinement of F^2^ against ALL reflections. The weighted R-factor wR and goodness of fit S are based on F^2^, conventional R-factors R are based on F, with F set to zero for negative F^2^. The threshold expression of F^2^ > 2sigma(F^2^) is used only for calculating R-factors(gt) etc. and is not relevant to the choice of reflections for refinement. R-factors based on F^2^ are statistically about twice as large as those based on F, and R- factors based on ALL data will be even larger.

### (2secBP2-SiPc) Bis(3-iodophenoxy)(phthalocyanine)silicon Fractional atomic coordinates and isotropic or equivalent isotropic displacement parameters (Å^2^)

**Table:**

	*x*	*y*	*z*	*U*_iso_*/*U*_eq_	
Si1	0.0000	0.2500	0.14722 (3)	0.0424 (3)	
O1	0.10423 (18)	0.30013 (7)	0.15037 (5)	0.0502 (5)	
N1	0.0000	0.2500	0.09000 (10)	0.0477 (8)	
N2	0.1543 (2)	0.18453 (10)	0.07595 (7)	0.0557 (6)	
N3	0.1325 (2)	0.20136 (9)	0.14701 (6)	0.0469 (6)	
N4	0.1747 (2)	0.19492 (9)	0.21844 (7)	0.0467 (6)	
N5	0.0000	0.2500	0.20454 (8)	0.0414 (7)	
C1	0.0427 (3)	0.22952 (14)	−0.04755 (9)	0.0655 (9)	
H1	0.0707	0.2161	−0.0722	0.079*	
C2	0.0858 (3)	0.20892 (13)	−0.01231 (8)	0.0600 (8)	
H2	0.1428	0.1816	−0.0123	0.072*	
C3	0.0421 (3)	0.22993 (12)	0.02363 (8)	0.0527 (7)	
C4	0.0689 (3)	0.21830 (11)	0.06512 (8)	0.0484 (7)	
C5	0.1844 (3)	0.17777 (11)	0.11392 (8)	0.0535 (7)	
C6	0.2850 (3)	0.14513 (12)	0.12648 (9)	0.0586 (8)	
C7	0.3677 (3)	0.11427 (14)	0.10481 (11)	0.0758 (11)	
H7A	0.3629	0.1112	0.0767	0.091*	
C8	0.4562 (4)	0.08877 (15)	0.12662 (12)	0.0847 (12)	
H8A	0.5136	0.0681	0.1129	0.102*	
C9	0.4635 (4)	0.09263 (15)	0.16849 (12)	0.0799 (11)	
H9A	0.5243	0.0740	0.1823	0.096*	
C10	0.3830 (3)	0.12339 (12)	0.18996 (10)	0.0623 (8)	
H10A	0.3883	0.1265	0.2181	0.075*	
C11	0.2936 (3)	0.14950 (11)	0.16799 (9)	0.0536 (7)	
C12	0.1969 (2)	0.18421 (11)	0.18043 (8)	0.0473 (6)	
C13	0.0820 (2)	0.22489 (10)	0.22928 (8)	0.0433 (6)	
C14	0.0520 (2)	0.23430 (10)	0.27105 (8)	0.0447 (6)	
C15	0.1064 (3)	0.21848 (12)	0.30650 (8)	0.0540 (7)	
H15	0.1773	0.1978	0.3065	0.065*	
C16	0.0525 (3)	0.23430 (13)	0.34196 (9)	0.0597 (8)	
H16	0.0867	0.2238	0.3666	0.072*	
C17	0.1460 (3)	0.33683 (12)	0.12375 (9)	0.0561 (7)	
C18	0.2136 (3)	0.32242 (15)	0.09020 (9)	0.0658 (9)	
H18A	0.2303	0.2871	0.0854	0.079*	
C19	0.2570 (4)	0.3601 (2)	0.06368 (12)	0.0995 (15)	
H19A	0.3009	0.3505	0.0405	0.119*	
C20	0.2341 (5)	0.4119 (2)	0.07211 (19)	0.123 (2)	
H20A	0.2588	0.4378	0.0538	0.147*	
C21	0.1763 (5)	0.42528 (19)	0.10666 (19)	0.126 (2)	
H21A	0.1681	0.4607	0.1130	0.151*	
C22	0.1292 (4)	0.38908 (15)	0.13276 (15)	0.0906 (13)	
C23	0.0718 (5)	0.40626 (17)	0.17334 (18)	0.128 (2)	
H23A	0.0410	0.3745	0.1867	0.154*	
C24	0.1738 (8)	0.4300 (3)	0.20158 (18)	0.179 (3)	
H24A	0.2376	0.4044	0.2059	0.269*	
H24B	0.1377	0.4395	0.2272	0.269*	
H24C	0.2085	0.4606	0.1890	0.269*	
C25	−0.0369 (6)	0.4412 (2)	0.1666 (3)	0.181 (4)	
H25A	−0.0956	0.4249	0.1482	0.218*	
H25B	−0.0110	0.4745	0.1551	0.218*	
C26	−0.0956 (8)	0.4494 (3)	0.2095 (3)	0.226 (4)	
H26A	−0.1689	0.4704	0.2070	0.339*	
H26B	−0.0374	0.4668	0.2269	0.339*	
H26C	−0.1168	0.4159	0.2210	0.339*	

### (2secBP2-SiPc) Bis(3-iodophenoxy)(phthalocyanine)silicon Atomic displacement parameters (Å^2^)

**Table:**

	*U*^11^	*U*^22^	*U*^33^	*U*^12^	*U*^13^	*U*^23^
Si1	0.0459 (6)	0.0491 (6)	0.0323 (5)	0.0052 (4)	0.000	0.000
O1	0.0573 (12)	0.0531 (11)	0.0402 (11)	−0.0034 (9)	−0.0045 (8)	0.0066 (8)
N1	0.0552 (19)	0.0534 (18)	0.0344 (16)	0.0052 (14)	0.000	0.000
N2	0.0658 (15)	0.0622 (14)	0.0390 (13)	0.0115 (12)	0.0092 (11)	0.0023 (11)
N3	0.0516 (13)	0.0526 (13)	0.0366 (12)	0.0080 (10)	0.0062 (10)	0.0035 (9)
N4	0.0455 (12)	0.0558 (13)	0.0387 (12)	0.0043 (10)	0.0028 (10)	0.0038 (9)
N5	0.0434 (16)	0.0514 (17)	0.0295 (15)	0.0019 (13)	0.000	0.000
C1	0.066 (2)	0.097 (2)	0.0342 (15)	0.0023 (16)	0.0047 (14)	−0.0033 (15)
C2	0.0594 (18)	0.080 (2)	0.0410 (16)	0.0041 (15)	0.0065 (14)	−0.0070 (14)
C3	0.0555 (16)	0.0632 (17)	0.0395 (15)	−0.0014 (13)	−0.0014 (13)	−0.0007 (12)
C4	0.0522 (15)	0.0568 (16)	0.0363 (14)	0.0049 (13)	0.0058 (12)	−0.0003 (11)
C5	0.0628 (18)	0.0565 (16)	0.0411 (16)	0.0103 (14)	0.0108 (13)	0.0017 (12)
C6	0.0665 (19)	0.0603 (17)	0.0490 (17)	0.0188 (14)	0.0146 (14)	0.0095 (13)
C7	0.091 (3)	0.077 (2)	0.059 (2)	0.032 (2)	0.0247 (18)	0.0101 (17)
C8	0.088 (3)	0.080 (2)	0.086 (3)	0.039 (2)	0.024 (2)	0.016 (2)
C9	0.075 (2)	0.080 (2)	0.084 (3)	0.034 (2)	0.007 (2)	0.0160 (19)
C10	0.0606 (18)	0.0678 (19)	0.0586 (18)	0.0153 (15)	0.0047 (15)	0.0135 (15)
C11	0.0535 (16)	0.0553 (16)	0.0518 (18)	0.0100 (13)	0.0095 (13)	0.0102 (13)
C12	0.0447 (14)	0.0535 (15)	0.0437 (15)	0.0061 (12)	0.0029 (12)	0.0086 (12)
C13	0.0395 (13)	0.0524 (14)	0.0379 (14)	−0.0013 (11)	−0.0006 (11)	0.0006 (11)
C14	0.0406 (13)	0.0557 (15)	0.0379 (14)	−0.0030 (11)	0.0018 (11)	0.0012 (11)
C15	0.0486 (15)	0.0730 (19)	0.0403 (15)	0.0000 (13)	−0.0051 (13)	0.0035 (13)
C16	0.0605 (18)	0.082 (2)	0.0365 (14)	−0.0010 (15)	−0.0072 (14)	0.0028 (14)
C17	0.0517 (16)	0.0635 (18)	0.0530 (17)	−0.0010 (13)	0.0000 (14)	0.0167 (14)
C18	0.0513 (17)	0.098 (2)	0.0487 (18)	−0.0062 (16)	−0.0014 (14)	0.0108 (17)
C19	0.072 (2)	0.159 (5)	0.068 (3)	−0.022 (3)	0.008 (2)	0.037 (3)
C20	0.117 (4)	0.122 (4)	0.129 (4)	−0.012 (3)	0.013 (3)	0.076 (4)
C21	0.127 (4)	0.085 (3)	0.164 (5)	0.008 (3)	0.054 (4)	0.058 (3)
C22	0.092 (3)	0.059 (2)	0.120 (3)	0.000 (2)	0.033 (2)	0.022 (2)
C23	0.155 (5)	0.059 (2)	0.171 (5)	−0.016 (3)	0.082 (4)	−0.007 (3)
C24	0.259 (8)	0.144 (5)	0.135 (5)	−0.085 (6)	0.066 (5)	−0.030 (4)
C25	0.143 (5)	0.085 (3)	0.316 (9)	−0.011 (4)	0.099 (6)	−0.056 (5)
C26	0.183 (7)	0.172 (7)	0.324 (11)	0.024 (6)	0.067 (8)	−0.090 (8)

### (2secBP2-SiPc) Bis(3-iodophenoxy)(phthalocyanine)silicon Geometric parameters (Å, º)

**Table:**

Si1—O1	1.7236 (19)	C10—C11	1.391 (4)
Si1—O1^i^	1.7237 (19)	C10—H10A	0.9400
Si1—N1	1.900 (3)	C11—C12	1.444 (4)
Si1—N5	1.904 (3)	C13—C14	1.446 (4)
Si1—N3	1.913 (2)	C14—C15	1.380 (4)
Si1—N3^i^	1.913 (2)	C14—C14^i^	1.394 (5)
O1—C17	1.372 (3)	C15—C16	1.378 (4)
N1—C4^i^	1.384 (3)	C15—H15	0.9400
N1—C4	1.384 (3)	C16—C16^i^	1.403 (6)
N2—C5	1.315 (4)	C16—H16	0.9400
N2—C4	1.324 (4)	C17—C18	1.387 (4)
N3—C5	1.378 (3)	C17—C22	1.390 (5)
N3—C12	1.386 (3)	C18—C19	1.392 (5)
N4—C12	1.314 (4)	C18—H18A	0.9400
N4—C13	1.323 (3)	C19—C20	1.385 (8)
N5—C13^i^	1.376 (3)	C19—H19A	0.9400
N5—C13	1.376 (3)	C20—C21	1.354 (7)
C1—C2	1.368 (4)	C20—H20A	0.9400
C1—C1^i^	1.408 (7)	C21—C22	1.372 (6)
C1—H1	0.9400	C21—H21A	0.9400
C2—C3	1.394 (4)	C22—C23	1.550 (7)
C2—H2	0.9400	C23—C25	1.506 (9)
C3—C3^i^	1.383 (6)	C23—C24	1.578 (7)
C3—C4	1.440 (4)	C23—H23A	0.9900
C5—C6	1.445 (4)	C24—H24A	0.9700
C6—C11	1.386 (4)	C24—H24B	0.9700
C6—C7	1.401 (4)	C24—H24C	0.9700
C7—C8	1.375 (5)	C25—C26	1.577 (7)
C7—H7A	0.9400	C25—H25A	0.9800
C8—C9	1.396 (6)	C25—H25B	0.9800
C8—H8A	0.9400	C26—H26A	0.9700
C9—C10	1.381 (5)	C26—H26B	0.9700
C9—H9A	0.9400	C26—H26C	0.9700
			
O1—Si1—O1^i^	173.03 (13)	C10—C11—C12	131.4 (3)
O1—Si1—N1	93.48 (7)	N4—C12—N3	127.5 (2)
O1^i^—Si1—N1	93.49 (7)	N4—C12—C11	122.6 (2)
O1—Si1—N5	86.52 (7)	N3—C12—C11	109.8 (2)
O1^i^—Si1—N5	86.51 (7)	N4—C13—N5	127.6 (2)
N1—Si1—N5	180.0	N4—C13—C14	122.2 (2)
O1—Si1—N3	89.42 (10)	N5—C13—C14	110.3 (2)
O1^i^—Si1—N3	90.61 (10)	C15—C14—C14^i^	121.46 (17)
N1—Si1—N3	89.79 (7)	C15—C14—C13	132.2 (3)
N5—Si1—N3	90.21 (7)	C14^i^—C14—C13	106.38 (15)
O1—Si1—N3^i^	90.61 (10)	C16—C15—C14	117.2 (3)
O1^i^—Si1—N3^i^	89.42 (10)	C16—C15—H15	121.4
N1—Si1—N3^i^	89.79 (7)	C14—C15—H15	121.4
N5—Si1—N3^i^	90.21 (7)	C15—C16—C16^i^	121.30 (18)
N3—Si1—N3^i^	179.58 (14)	C15—C16—H16	119.3
C17—O1—Si1	134.08 (18)	C16^i^—C16—H16	119.3
C4^i^—N1—C4	106.7 (3)	O1—C17—C18	120.7 (3)
C4^i^—N1—Si1	126.66 (15)	O1—C17—C22	118.9 (3)
C4—N1—Si1	126.66 (15)	C18—C17—C22	120.1 (3)
C5—N2—C4	121.6 (2)	C17—C18—C19	120.2 (4)
C5—N3—C12	106.8 (2)	C17—C18—H18A	119.9
C5—N3—Si1	127.05 (19)	C19—C18—H18A	119.9
C12—N3—Si1	126.11 (18)	C20—C19—C18	118.7 (4)
C12—N4—C13	121.6 (2)	C20—C19—H19A	120.6
C13^i^—N5—C13	106.7 (3)	C18—C19—H19A	120.6
C13^i^—N5—Si1	126.63 (14)	C21—C20—C19	120.0 (4)
C13—N5—Si1	126.63 (14)	C21—C20—H20A	120.0
C2—C1—C1^i^	121.19 (19)	C19—C20—H20A	120.0
C2—C1—H1	119.4	C20—C21—C22	122.5 (5)
C1^i^—C1—H1	119.4	C20—C21—H21A	118.8
C1—C2—C3	117.7 (3)	C22—C21—H21A	118.8
C1—C2—H2	121.2	C21—C22—C17	118.1 (4)
C3—C2—H2	121.2	C21—C22—C23	120.5 (4)
C3^i^—C3—C2	121.13 (18)	C17—C22—C23	121.1 (3)
C3^i^—C3—C4	106.89 (16)	C25—C23—C22	111.1 (5)
C2—C3—C4	132.0 (3)	C25—C23—C24	114.5 (5)
N2—C4—N1	127.4 (2)	C22—C23—C24	109.9 (4)
N2—C4—C3	122.6 (2)	C25—C23—H23A	107.0
N1—C4—C3	109.8 (2)	C22—C23—H23A	107.0
N2—C5—N3	127.1 (3)	C24—C23—H23A	107.0
N2—C5—C6	123.0 (3)	C23—C24—H24A	109.5
N3—C5—C6	109.8 (2)	C23—C24—H24B	109.5
C11—C6—C7	120.9 (3)	H24A—C24—H24B	109.5
C11—C6—C5	106.9 (2)	C23—C24—H24C	109.5
C7—C6—C5	132.2 (3)	H24A—C24—H24C	109.5
C8—C7—C6	116.9 (3)	H24B—C24—H24C	109.5
C8—C7—H7A	121.5	C23—C25—C26	105.4 (6)
C6—C7—H7A	121.5	C23—C25—H25A	110.7
C7—C8—C9	122.0 (3)	C26—C25—H25A	110.7
C7—C8—H8A	119.0	C23—C25—H25B	110.7
C9—C8—H8A	119.0	C26—C25—H25B	110.7
C10—C9—C8	121.3 (3)	H25A—C25—H25B	108.8
C10—C9—H9A	119.4	C25—C26—H26A	109.5
C8—C9—H9A	119.4	C25—C26—H26B	109.5
C9—C10—C11	116.9 (3)	H26A—C26—H26B	109.5
C9—C10—H10A	121.6	C25—C26—H26C	109.5
C11—C10—H10A	121.6	H26A—C26—H26C	109.5
C6—C11—C10	122.0 (3)	H26B—C26—H26C	109.5
C6—C11—C12	106.6 (2)		

Symmetry code: (i) −*x*, −*y*+1/2, *z*.
