# Supplementary material for: Crystal structures of bis­(phen­oxy)silicon phthalocyanines: increasing π–π inter­actions, solubility and disorder and no halogen bonding observed
Source: Acta Crystallogr E Crystallogr Commun. 2016 Jun 21;72(Pt 7):988–94. doi: 10.1107/S205698901600935X (PMC4992922; doi:10.1107/S205698901600935X)
Supplement: Supplementary file 5 [file e-72-00988-sup5.pdf]

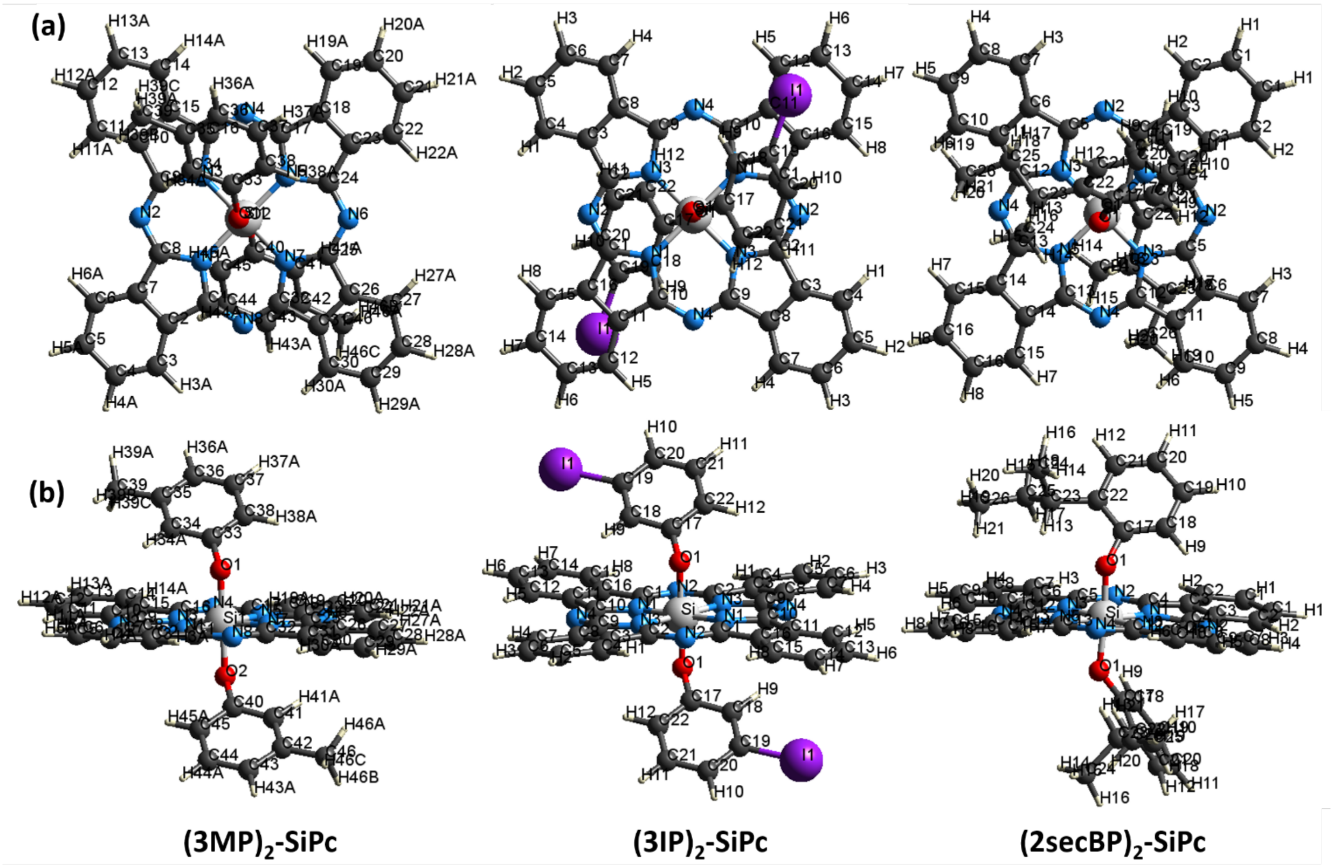

Scheme S1. – Labeled crystal structure organizations of $(3MP)_2$-SiPc (left), $(3IP)_2$-SiPc (middle) and $(2secBP)_2$-SiPc (right), obtained by single crystal x-ray diffraction where A) top view and B) side view.
