# Supplementary figures and images for: Crystal structures of bis­(phen­oxy)silicon phthalocyanines: increasing π–π inter­actions, solubility and disorder and no halogen bonding observed
Source: Acta Crystallogr E Crystallogr Commun. 2016 Jun 21;72(Pt 7):988–94. doi: 10.1107/S205698901600935X (PMC4992922; doi:10.1107/S205698901600935X)

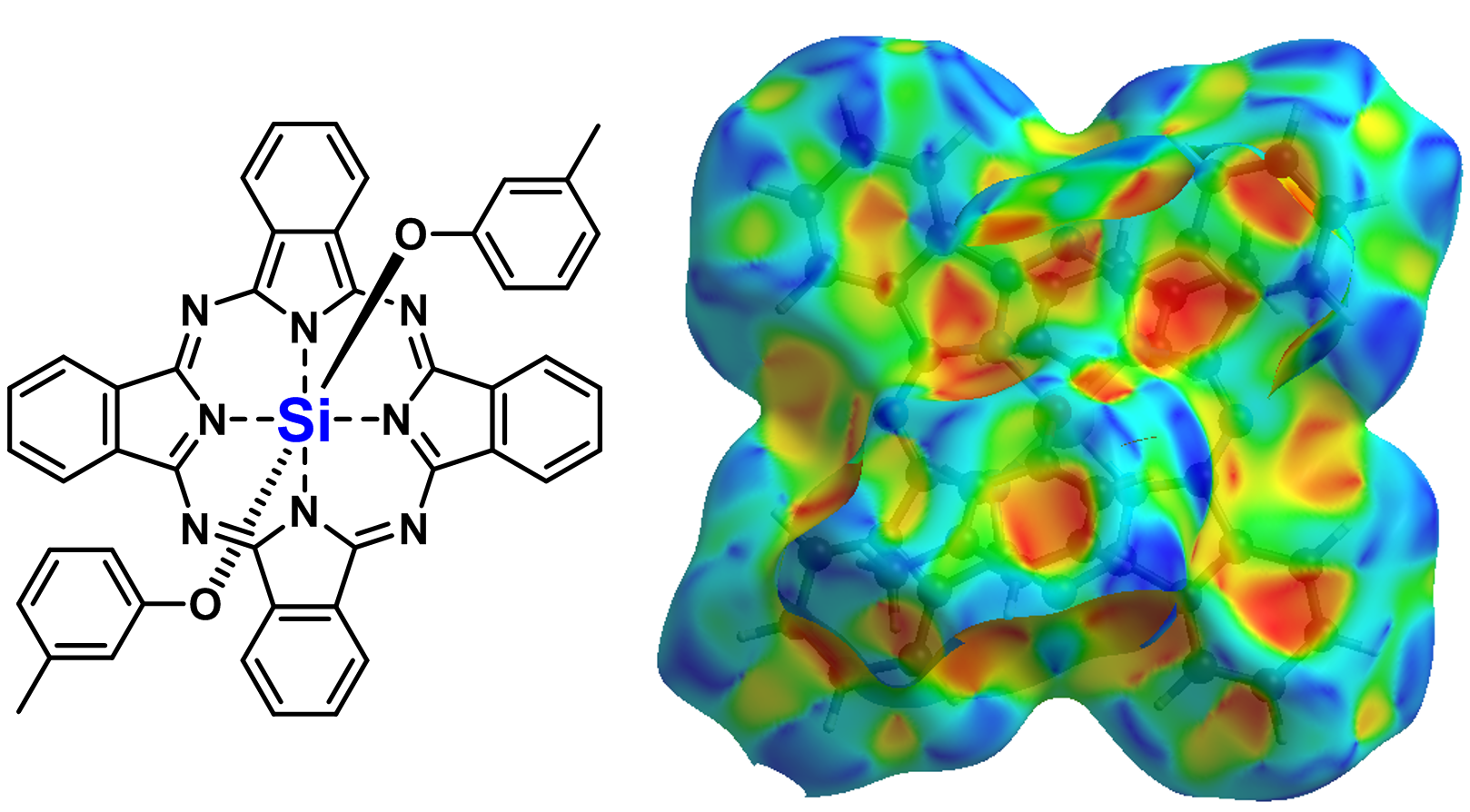

Supplement: Supplementary file 6 [file e-72-00988-sup6.tif]

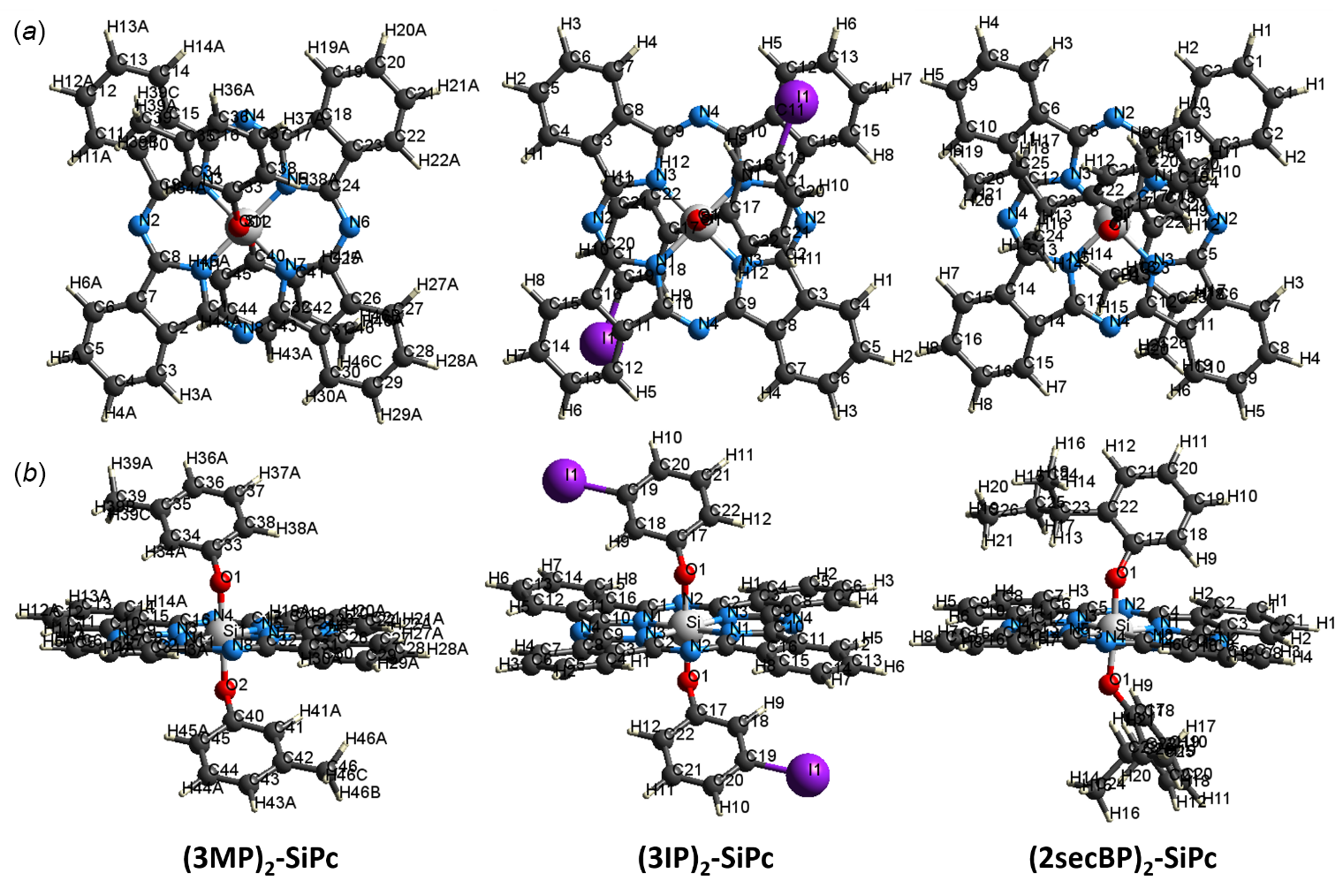

Supplement: Supplementary file 7 [file e-72-00988-sup7.tif]
